# BPDCN MYB fusions regulate cell cycle genes, impair differentiation, and induce myeloid–dendritic cell leukemia

**DOI:** 10.1172/jci.insight.183889

**Published:** 2024-12-20

**Authors:** Christopher A.G. Booth, Juliette M. Bouyssou, Katsuhiro Togami, Olivier Armand, Hembly G. Rivas, Kezhi Yan, Siobhan Rice, Shuyuan Cheng, Emily M. Lachtara, Jean-Pierre Bourquin, Alex Kentsis, Esther Rheinbay, James A. DeCaprio, Andrew A. Lane

**Affiliations:** 1 Department of Medical Oncology, Dana-Farber Cancer Institute, Harvard Medical School, Boston, Massachusetts, USA.; 2 Program in Virology, Graduate School of Arts and Sciences, Harvard University, Cambridge, Massachusetts, USA.; 3 Tow Center for Developmental Oncology, Department of Pediatrics, Memorial Sloan Kettering Cancer Center, New York, New York, USA.; 4 Molecular Pharmacology Program, Sloan Kettering Institute, New York, New York, USA.; 5 Departments of Pediatrics, Pharmacology, and Physiology & Biophysics, Weill Medical College of Cornell University, New York, New York, USA.; 6 Krantz Family Center for Cancer Research, Boston, Massachusetts, USA; 7 Broad Institute of MIT and Harvard, Cambridge, Massachusetts, USA.; 8 Division of Oncology, Children’s Research Center, University Children’s Hospital, Zurich, Switzerland.

**Keywords:** Hematology, Leukemias, Oncogenes

## Abstract

MYB fusions are recurrently found in select cancers, including blastic plasmacytoid DC neoplasm (BPDCN), an acute leukemia with poor prognosis. They are markedly enriched in BPDCN compared with other blood cancers and, in some patients, are the only obvious somatic mutation detected. This suggests that they may alone be sufficient to drive DC transformation. MYB fusions are hypothesized to alter the normal transcription factor activity of MYB, but, mechanistically, how they promote leukemogenesis is poorly understood. Using CUT&RUN chromatin profiling, we found that, in BPDCN leukemogenesis, MYB switches from being a regulator of DC lineage genes to aberrantly regulating G2/M cell cycle control genes. MYB fusions found in patients with BPDCN increased the magnitude of DNA binding at these locations, and this was linked to BPDCN-associated gene expression changes. Furthermore, expression of MYB fusions in vivo impaired DC differentiation and induced transformation to generate a mouse model of myeloid-dendritic acute leukemia. Therapeutically, we present evidence that all-trans retinoic acid (ATRA) may cause loss of MYB protein and cell death in BPDCN.

## Abstract 2

MYB fusions associated with BPDCN regulate cell cycling and promote myeloid-dendritic leukemia in vivo. All-trans retinoic acid causes loss of MYB in BPDCN cells.

## Graphical abstract

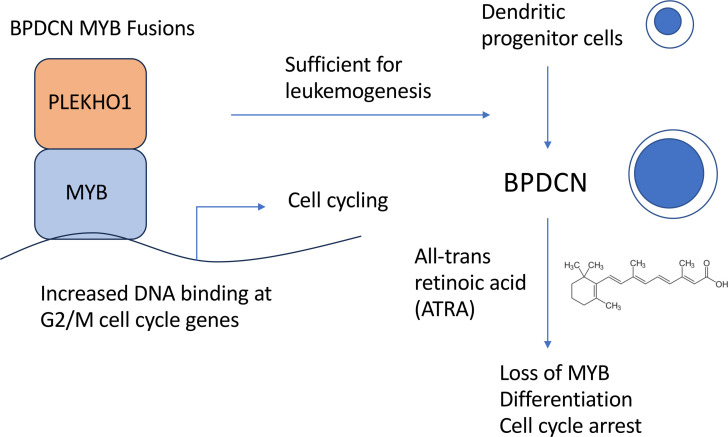

## Introduction

Rearrangements of the hematopoietic transcription factor *MYB* are recurrently found in 20% of patients with blastic plasmacytoid DC neoplasm (BPDCN), a rare leukemia arising from cells of the plasmacytoid DC (pDC) lineage (1–4). These rearrangements result in fusion of the MYB N-terminus to the C-terminus of one of a number of partner proteins, including PLEKHO1, DCPS, and ZFAT (2). The *MYB::ZFAT* fusion is out of frame, resulting in expression of truncated MYB with no partner protein. Loss of the C-terminus of MYB is thought to increase its activity via a number of proposed mechanisms (2, 5, 6).

MYB fusions have been identified in a select number of solid tumors, including adenoid cystic carcinoma (ACC) (7, 8) and angiocentric glioma (9). While expression of WT MYB is a dependency in many leukemias (10–13) and is sometimes associated with translocations that bring an active promoter into proximity of the *MYB* locus (14, 15), MYB fusions are very rare in non-BPDCN leukemias (4). Interestingly, in children, MYB fusions occur in up to 50% of patients and appear to require few or possibly no cooperating mutations to induce BPDCN (2). This suggests that, in some contexts, MYB fusions may be sufficient to induce leukemic transformation of pDC lineage cells. How this occurs, and why other hematopoietic lineages are not similarly affected, is unknown.

Globally, the mutational landscape of BPDCN shows similarities to both myeloid and lymphoid malignancies (4), which may reflect the lympho-myeloid ontogeny of pDCs (16, 17). Myeloid-type mutations in epigenetic regulators (such as *TET2*, *ASXL1*, and *EZH2*) and splicing factors (such as *ZRSR2* and *SRSF2*) are highly prevalent (18–20) and may arise from preleukemic clones (21). Other common BPDCN mutations, such as in the transcription factors *IKZF1* and *ETV6* (22, 23) and cell cycle regulator *CDKN2A* (22, 24–26), are normally associated with lymphoid malignancies. Disruption of the G1/S checkpoint via mutations in *CDKN2A* and/or *RB1* appears to be a hallmark of BPDCN (4, 24, 27). The *CDKN2A* gene encodes 2 proteins, p16INK4A and p14ARF (p19ARF in mouse), which together act to halt cell cycle progression in G1 phase through regulation of cyclin-dependent kinases and p53 (28, 29).

All-trans retinoic acid (ATRA) is a well-established treatment for acute promyelocytic leukemia (APL), which is induced by the fusion protein PML::RARA (30). In ACC, ATRA has been shown to cause downregulation of MYB expression, leading to reduced viability (31). Use of ATRA to downregulate MYB could, therefore, be a potential therapeutic approach for MYB-dependent leukemias, including BPDCN.

## Results

### MYB aberrantly regulates G2/M cell cycle genes in BPDCN.

**Figure 1:**
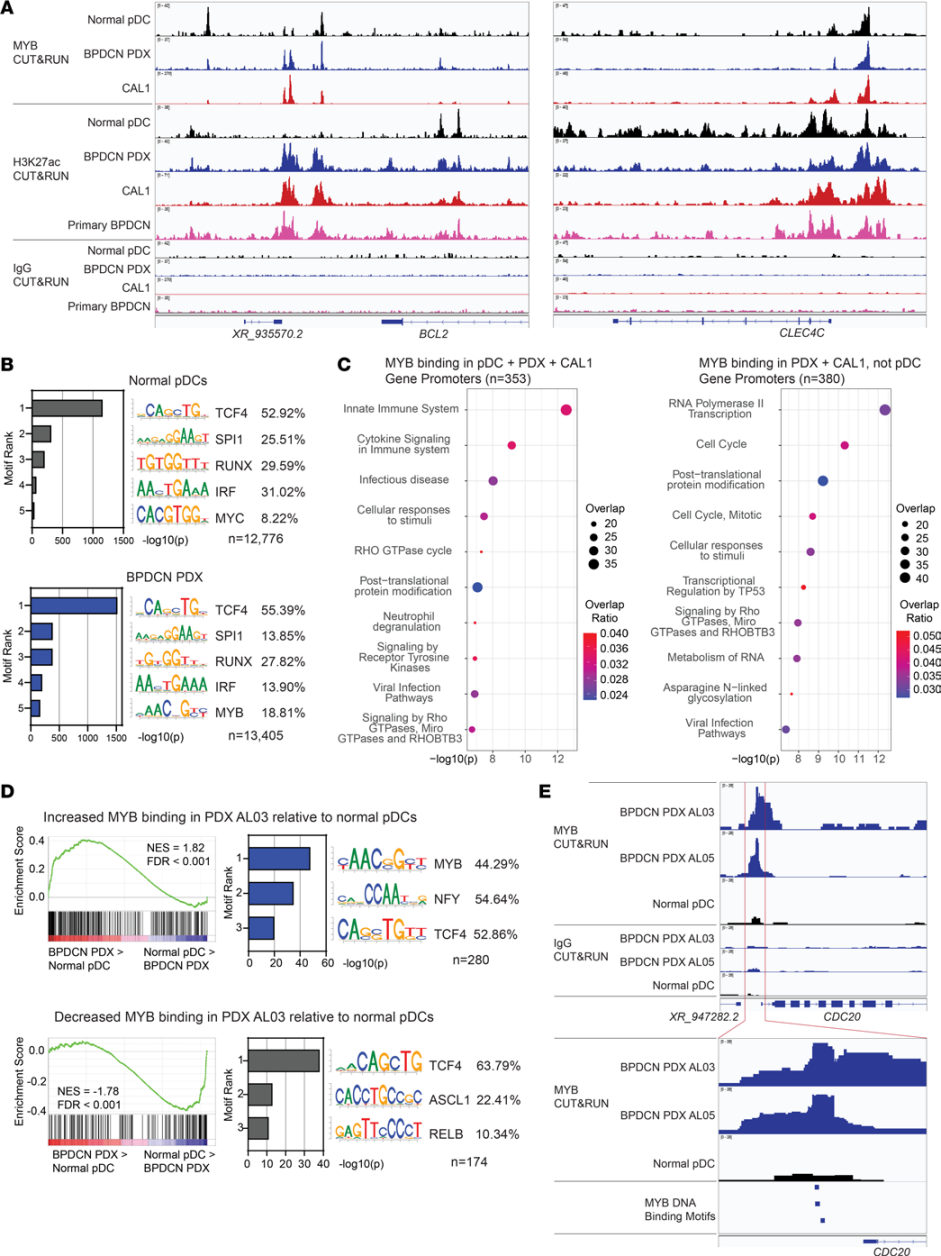
MYB aberrantly regulates G2/M cell cycle genes in BPDCN. (**A**) MYB, H3K27ac, and IgG CUT&RUN tracks at the *BCL2* and *CLEC4C* (BDCA2) loci in the indicated cell types. (**B**) Top 5 ranked motifs enriched in MYB CUT&RUN peaks in normal pDCs and BPDCN PDX cells, and percent of peaks containing each motif. (**C**) Top 10 ranked Reactome gene sets overlapping with genes showing promoter binding of MYB in the indicated samples. (**D**) (Left) GSEA comparing normal pDCs and BPDCN PDX cells for genes showing differential MYB binding at promoter regions in BPDCN PDX AL03 relative to normal pDCs (*n* = 283 genes increased and *n* = 181 genes decreased MYB binding). (Right) Top 3 ranked motifs enriched in differentially bound MYB sites mapping to promoters, and percent of sites containing each motif. (**E**) MYB and IgG CUT&RUN tracks at the *CDC20* locus in the indicated cell types.

To investigate the role of MYB in BPDCN, we performed CUT&RUN for MYB and the marker of active chromatin H3K27 acetylation (H3K27ac) in BPDCN cells and in normal pDCs. We used CUT&RUN rather than ChIP-Seq because of its lower cell input requirement (100,000 cells or fewer) (32) and the fact that normal pDCs are difficult to harvest from peripheral blood in sufficient quantities for ChIP-Seq. We observed similar chromatin profiles in primary patient BPDCN cells and in patient-derived xenografts (PDXs) from patients with BPDCN (Figure 1A and Supplemental Figure 1, A and B; supplemental material available online with this article; https://doi.org/10.1172/jci.insight.183889DS1). This facilitated more robust mechanistic analyses, as PDXs are a sustainable source of larger quantities of primary leukemia cells that phenocopy BPDCN cells harvested directly from patients (20, 33).

In normal pDCs, BPDCN PDXs, and the BPDCN cell line CAL1, we observed MYB binding and H3K27ac at pDC-lineage genes such as *CLEC4C* (BDCA2), as expected (Figure 1A). In contrast, at chromatin sites associated with oncogenes, such as at a *BCL2* 3′ enhancer, we observed increased MYB occupancy and H3K27ac in BPDCN cells compared with normal pDCs (Figure 1A), linking MYB to increased BCL2 expression in BPDCN (34).

We generated consensus MYB CUT&RUN peak sets for each sample type: normal pDCs (*n* = 16,018), BPDCN PDX (*n* = 17,806), and CAL1 (*n* = 17,055) (Supplemental Figure 1C and Supplemental Table 1). In all 3 cell types, MYB binding sites were strongly enriched for the DNA binding motif of TCF4, a critical transcriptional regulator of the pDC lineage (35, 36) (Figure 1B and Supplemental Figure 1D). In agreement with this, MYB binding sites in CAL1 cells showed strong overlap with TCF4 binding sites in CAL1 cells from a previously published ChIP-Seq dataset (36) (Supplemental Figure 1E). Canonical MYB DNA binding motifs were relatively weakly enriched compared with TCF4, SPI1, RUNX, and IRF motifs. This may indicate cooperative DNA binding with other transcription factors at lineage-related MYB binding sites in these cells.

To explore which genes were regulated by MYB in BPDCN and normal pDCs, we narrowed our analysis to binding sites located at gene promoters. Genes with promoters bound by MYB in CAL1, BPDCN PDX, and normal pDCs (*n* = 353) were enriched in Reactome gene sets related to the pDC lineage, such as “Innate Immune System” and “Viral Infection Pathways” (Figure 1C). Conversely, genes with promoters selectively bound by MYB in CAL1 and BPDCN PDX but not normal pDCs (*n* = 380) were enriched in the “Cell Cycle” and “Cell Cycle, Mitotic” gene sets, suggesting that MYB plays a role in direct regulation of cell cycle genes in malignant BPDCN, particularly those active in the G2/M checkpoint (37).

We then performed genome-wide DiffBind analysis to determine all regions significantly differentially bound by MYB in BPDCN relative to normal pDCs (Supplemental Table 2). We used 2 PDX samples derived from separate patients with BPDCN, one of which (AL05) carried a MYB::ZFAT fusion (Supplemental Figure 1F) and compared each to normal pDCs from 3 healthy donors. In both PDX samples, sites that were significantly more strongly bound by MYB in BPDCN compared with normal pDCs and located at promoter regions showed increased expression in BPDCN relative to normal pDCs (Figure 1D and Supplemental Figure 1G) (20). These included cell cycle regulators such as *CDC20* and *CCP110* (Figure 1E and Supplemental Figure 1H) and were enriched for MYB binding motifs (Figure 1D and Supplemental Figure 1G). Conversely, sites significantly more strongly bound in pDCs compared with BPDCN showed decreased expression in BPDCN relative to normal pDCs and were enriched for TCF4 motifs (Figure 1D and Supplemental Figure 1G). These findings were true for both BPDCN PDX samples and implicate MYB binding at cell cycle gene promoters containing MYB binding motifs in BPDCN transformation.

CUT&RUN enables high-resolution localization of transcription factor binding sites using footprint analysis (38). We performed this analysis for MYB binding in CAL1 cells and, for each of the DNA motifs identified at MYB binding sites in CAL1 (Supplemental Figure 1D), identified CUT&RUN footprints containing these motifs. Compared with MYB CUT&RUN footprints containing TCF4, RUNX, IRF, or SPI1 motifs, footprints containing MYB motifs were more likely to be located at promoter regions (Supplemental Figure 1I), suggesting a role for these binding sites in direct control of gene expression. Indeed, genes with MYB CUT&RUN footprints containing MYB motifs at their promoters were enriched for increased expression in BPDCN PDX cells relative to normal pDCs (Supplemental Figure 1I and Supplemental Table 3) (20). Conversely, genes with MYB CUT&RUN footprints containing TCF4, RUNX, IRF, or SPI1 motifs at their promoters were enriched for increased expression in normal pDCs. The 500 highest-confidence footprints containing TCF4 motifs and mapping to promoters were enriched at pDC lineage-associated genes, while the 324 footprints containing MYB motifs and mapping to promoters were enriched at cell cycle genes (Supplemental Figure 1J).

Together, these findings implicate MYB in regulation of pDC lineage genes in normal pDCs via cooperative DNA binding with other transcription factors, particularly TCF4. Conversely, in BPDCN, MYB is a direct regulator of cell cycle genes which contain MYB DNA binding motifs at their promoters. We hypothesized that one of the key functions of MYB in driving BPDCN is by direct binding to DNA at cell cycle gene promoters via its DNA-binding domain. This aberrant binding could occur in BPDCNs with or without MYB fusions, since the N-terminal DNA-binding domain is retained in BPDCN MYB fusions (2, 3). Aberrant MYB binding could cause dysregulated expression of these genes, leading to loss of cell cycle control and contributing to BPDCN pathogenesis, since cycling is normally inhibited in dendritic progenitors undergoing terminal pDC differentiation (17).

### BPDCN MYB fusions cause increased binding to G2/M cell cycle genes.

**Figure 2:**
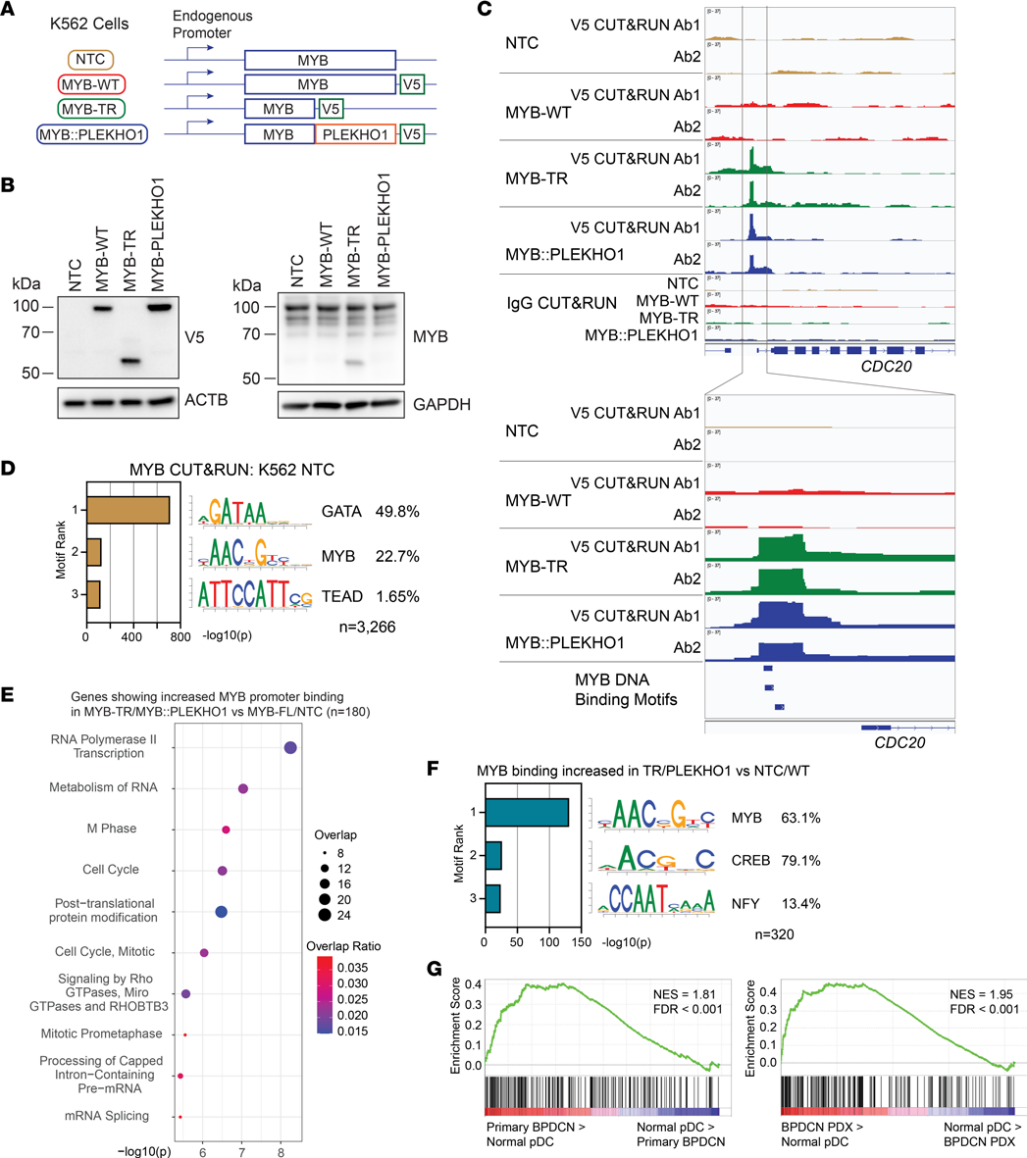
BPDCN MYB fusions cause increased binding to G2/M cell cycle genes. (**A**) Schematic showing expression of MYB-V5–knock-in constructs from the endogenous MYB locus in K562 cells. (**B**) Western blots showing expression of MYB-V5–knock-in constructs in K562 cells. (**C**) V5 and IgG CUT&RUN tracks at the *CDC20* locus in MYB-V5–knock-in K562 cells of the indicated genotypes, for 2 different V5 antibodies. (**D**) Top 3 ranked motifs enriched in MYB CUT&RUN peaks in K562 nontransfected control (NTC) cells, and percent of peaks containing each motif. (**E**) Top 10 ranked Reactome gene sets overlapping with genes showing differentially increased MYB CUT&RUN binding in MYB-TR and MYB::PLEKHO1 relative to NTC and MYB-WT K562 cells at promoter regions. (**F**) Top 3 motifs enriched in MYB CUT&RUN peaks showing differentially increased binding in MYB-TR and MYB::PLEKHO1 relative to NTC and MYB-WT K562 cells, and percent of peaks containing each motif. (**G**) GSEA comparing (left) normal pDCs and primary BPDCN cells or (right) normal pDCs and BPDCN PDX cells for genes showing differentially increased MYB CUT&RUN binding at promoter regions in MYB-TR and MYB::PLEKHO1 relative to NTC and MYB-WT K562 cells (*n* = 180 genes).

Since MYB fusions are the only detectable mutations in some patients with BPDCN (2), we wondered whether MYB fusions are sufficient to cause increased binding of MYB at G2/M cell cycle genes. To first investigate this in a cellular context independent of the pDC lineage and using endogenous MYB expression levels, we knocked in V5-tagged MYB constructs to the endogenous MYB locus of the acute myeloid leukemia (AML) cell line K562 (Figure 2, A and B). This allows direct comparison of chromatin binding between WT MYB (MYB-WT) and BPDCN-associated MYB fusions. CUT&RUN for V5 revealed a strong relative enrichment of truncated MYB (MYB-TR) and MYB::PLEKHO1 binding compared with MYB-WT binding at the transcription start sites of G2/M cell cycle regulators such as *CDC20* (Figure 2C) and *CDCA3* (Supplemental Figure 2A). These binding sites contained MYB DNA binding motifs (Figure 2C and Supplemental Figure 2A).

We next performed CUT&RUN using a MYB antibody that recognizes the N-terminus (retained in BPDCN MYB fusions), detecting both knocked-in MYB-V5 and endogenous MYB (Supplemental Figure 2B). MYB-bound sites were enriched for GATA motifs (Figure 2D), consistent with GATA factors nucleating transcriptional regulatory complexes in K562 cells (39). We performed genome-wide analysis to identify sites of differential MYB binding in MYB-TR and MYB::PLEKHO1–knock-in cells relative to MYB-WT–knock-in cells and nontransfected control (NTC) K562 cells (Supplemental Table 2). This revealed enriched binding of MYB-TR and MYB::PLEKHO1 at transcription start sites of G2/M cell cycle regulators including *CCP110* and *CENPM* (Figure 2E and Supplemental Figure 2C). In contrast, non–cell cycle genes expressed in K562 cells such as *ERMAP* and *CCRL2* did not show differential binding (Supplemental Figure 2C). Differentially bound regions were enriched for MYB motifs and not GATA motifs (Figure 2F), suggesting importance of the N-terminal domain of MYB-TR and MYB::PLEKHO1 in binding to DNA at these sites. These differentially bound genes were strongly enriched for expression in primary BPDCN or BPDCN PDX cells relative to normal pDCs (Figure 2G) (20).

These data, using endogenous MYB expression levels (Figure 2B), demonstrate that BPDCN MYB fusions increase the intrinsic binding of MYB to DNA specifically at G2/M cell cycle genes that are strongly expressed in BPDCN. Given that MYB fusions may independently cause BPDCN in some patients (2), we wondered whether this increased DNA binding is sufficient to induce leukemia in vivo.

### BPDCN MYB fusions impair differentiation in dendritic progenitor cells.

To address this, we generated Hoxb8-FL immortalized hematopoietic progenitor cells as previously described (40), from mice constitutively expressing Cas9 and GFP (41). Hoxb8-FL cells proliferate indefinitely in the presence of estradiol and FLT3 ligand (FLT3L), and undergo myeloid, lymphoid, and dendritic differentiation upon withdrawal of estradiol in vitro or transplantation in vivo (40). This system allows efficient lentiviral sgRNA KO or cDNA expression in nontransformed hematopoietic progenitor cells, followed by interrogation of differentiation potential using in vitro and in vivo assays (42, 43). Using Hoxb8-FL cells also allowed us to explore whether MYB or BPDCN MYB fusions could confer self-renewal specifically in hematopoietic progenitor cells, rather than targeting already self-renewing hematopoietic stem cells as would be the case using lentiviral transduction of primary bone marrow.

**Figure 3:**
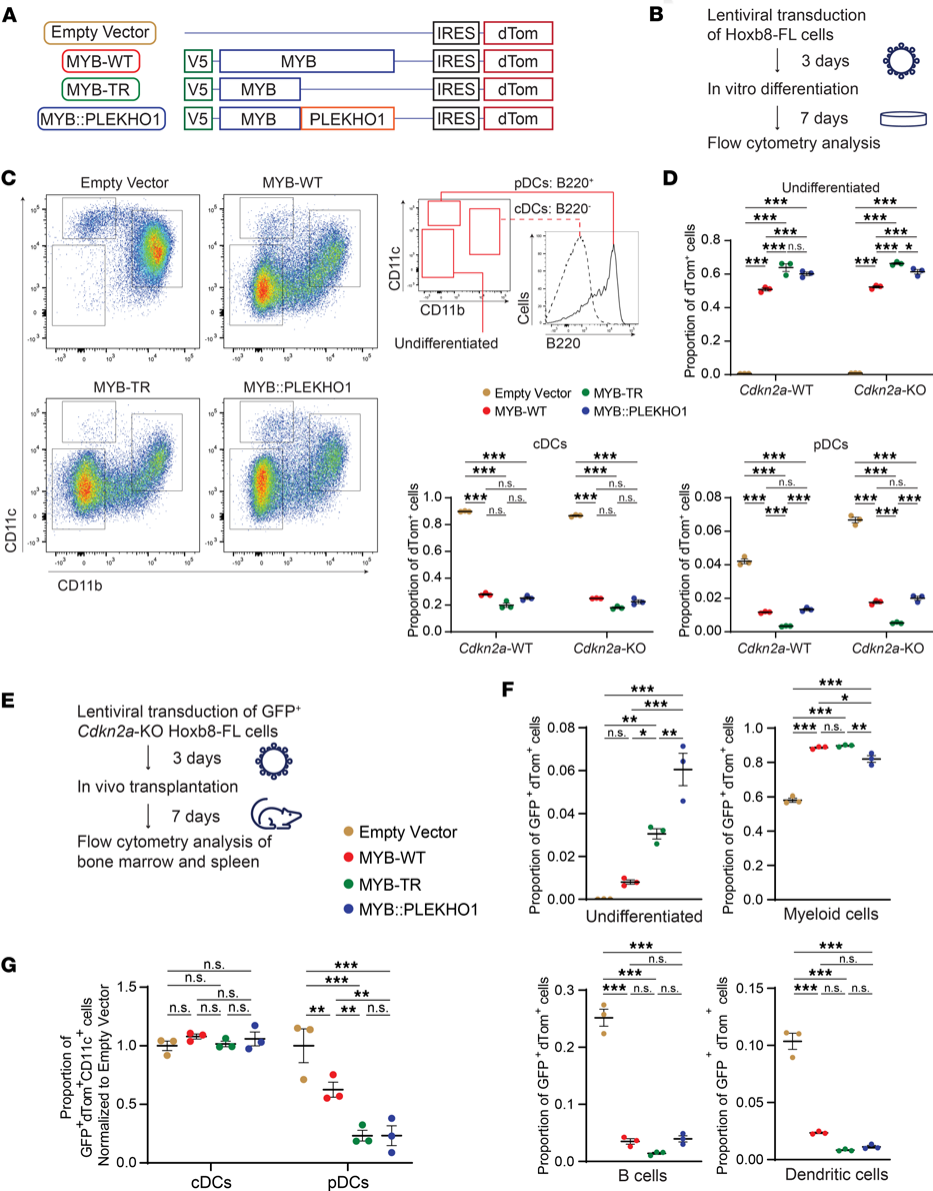
BPDCN MYB fusions impair differentiation in dendritic progenitor cells. (**A**) Schematic showing coexpression of V5-MYB constructs with a dTom reporter in Hoxb8-FL cells. (**B**) Experimental setup for Hoxb8-FL in vitro differentiation assays. (**C**) Representative flow cytometry plots at day 7 of in vitro differentiation of Hoxb8-FL cells of the indicated genotypes. Histogram on right shows B220 expression in empty vector cDCs and pDCs. (**D**) Number of dTom^+^ cells of the indicated cell types as a proportion of total dTom^+^ cells (*n* = 3 for each genotype). CD11b^–^CD11c^–^B220^–^ undifferentiated cells, CD11b^+^CD11c^+^B220^–^ cDCs, and CD11b^–^CD11c^+^B220^+^ pDCs. (**E**) Experimental setup for Hoxb8-FL in vivo differentiation assays. (**F**) Number of GFP^+^dTom^+^ bone marrow cells of the indicated cell types as a proportion of total GFP^+^dTom^+^ bone marrow cells (*n* = 3 for each genotype). CD19^–^CD11c^–^CD11b^–^B220^–^ undifferentiated cells, CD11c^–^CD19^–^CD11b^+^ myeloid cells, CD11c^–^CD19^+^CD11b^–^B220^+^ B cells, and CD11c^+^CD19^–^ DCs. (**G**) Number of GFP^+^dTom^+^ spleen cells of the indicated cell types as a proportion of total GFP^+^dTom^+^CD11c^+^ spleen cells, normalized to empty vector (*n* = 3 for each genotype). CD11c^+^CD19^–^CD11b^+^B220^–^ cDCs and CD11c^+^CD19^–^CD11b^–^B220^+^BST2^+^SIGLEC-H^+^ pDCs. (**D, F,** and **G**) Data represent mean ± SEM. Significance determined by 1-way ANOVA with Tukey correction for multiple comparisons. *P < 0.05, **P < 0.01, ***P < 0.001.

To first investigate the effect of BPDCN MYB fusions on DC differentiation, we coexpressed a fluorescent dTom reporter with V5-tagged MYB-WT, MYB-TR, or MYB::PLEKHO1 in Hoxb8-FL cells using lentiviral transduction (Figure 3A and Supplemental Figure 3A). Empty vector–transduced (EV-transduced) cells, expressing only the dTom reporter, were used as controls. Since deletion of the G1/S cell cycle regulator *CDKN2A* is a common event in BPDCN (22, 24–26), we performed these experiments using either *Cdkn2a*-WT or *Cdkn2a*-KO Hoxb8-FL cells (Supplemental Figure 3, B and C).

We induced DC differentiation by withdrawal of estradiol and culture with FLT3L for 7 days (40) (Figure 3B). At day 7, all EV-transduced dTom^+^ cells showed surface expression of B220, CD11b, and/or CD11c, indicating conventional DC (cDC; CD11b^+^CD11c^+^B220^–^) or pDC (CD11b^–^CD11c^+^B220^+^) differentiation (Figure 3, C and D). A large proportion (50%–65%) of MYB-WT–, MYB-TR–, and MYB::PLEKHO1-transduced dTom^+^ cells, however, remained undifferentiated (CD11b^–^CD11c^–^B220^–^). This indicates impaired DC differentiation induced by exogenous expression of MYB. KO of *Cdkn2a* caused slightly increased numbers of pDCs (Figure 3D) but, otherwise, had little effect in this short-term differentiation assay.

We next interrogated in vivo differentiation potential by analyzing bone marrow and spleen of recipient mice 7 days after i.v. transplantation of *Cdkn2a*-KO Hoxb8-FL cells (Figure 3E). EV-transduced GFP^+^dTom^+^ cells mainly gave rise to myeloid cells (CD11b^+^CD11c^–^) and B cells (CD19^+^B220^+^) but also DCs (CD11c^+^) (Supplemental Figure 3D). Though rare, Hoxb8-FL–derived GFP^+^dTom^+^ pDCs (CD11b^–^CD11c^+^B220^+^SIGLEC-H^+^BST2^+^) were detectable in recipient bone marrow and spleen (Supplemental Figure 3E). Compared with EV, expression of all forms of MYB caused increased myeloid cells and reduced B cells and DCs as a proportion of total GFP^+^dTom^+^ cells in the bone marrow (Figure 3F) and spleen (Supplemental Figure 3F) of recipient mice. Similarly to our in vitro data, we observed a retention of GFP^+^dTom^+^ undifferentiated lineage-negative cells in MYB-WT, MYB-TR, and MYB::PLEKHO1, but not EV, recipient mice in the bone marrow (Figure 3F) and spleen (Supplemental Figure 3F). This persistence of GFP^+^dTom^+^ undifferentiated cells was most pronounced in MYB-TR and MYB::PLEKHO1 recipients. Furthermore, when looking at GFP^+^dTom^+^CD11c^+^ DCs in the spleen, MYB-TR and MYB::PLEKHO1 displayed a specific impairment in terminal pDC, but not cDC, differentiation compared with EV and MYB-WT (Figure 3G).

These data show that, when expressed in hematopoietic progenitor cells, BPDCN MYB fusions induce a specific impairment in pDC differentiation and retention of undifferentiated cells in vivo at 7 days after transplant. We next asked whether these undifferentiated cells would persist long-term and induce leukemia in recipient mice.

### BPDCN MYB fusions expressed in hematopoietic progenitor cells induce myeloid-dendritic cell leukemia in vivo.

**Figure 4:**
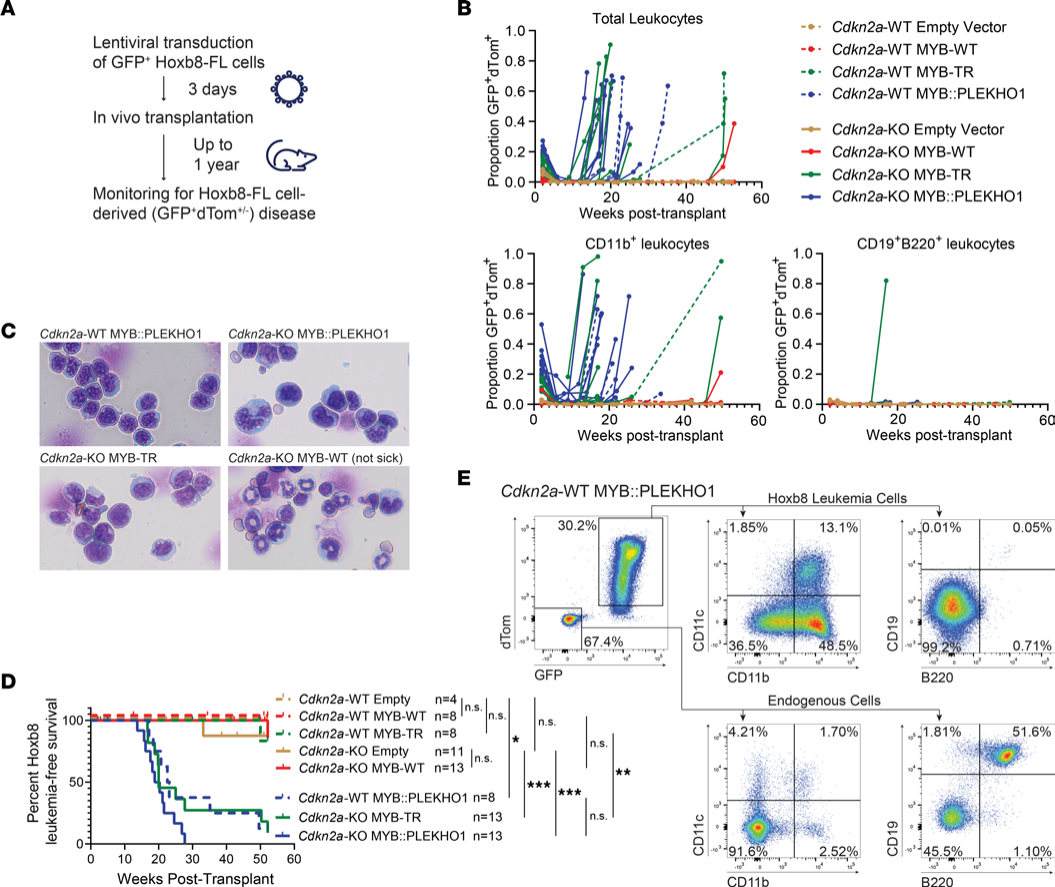
BPDCN MYB fusions expressed in hematopoietic progenitor cells induce myeloid-dendritic cell leukemia in vivo. (**A**) Experimental setup for Hoxb8-FL long-term in vivo follow-up. (**B**) GFP^+^dTom^+^ leukocytes as a proportion of total leukocytes of the indicated populations in mice transplanted with Hoxb8-FL cells of the indicated genotypes. (**C**) Representative H&E stains of bone marrow cells from 3 leukemic recipient mice and 1 healthy recipient mouse. Magnification, 40×. (**D**) Hoxb8-FL cell–derived leukemia-free survival curves for recipient mice. Significance determined by log-rank test. *P < 0.05, **P < 0.01, ***P < 0.001. (**E**) Peripheral blood flow cytometry plots for a leukemic *Cdkn2a*-WT MYB::PLEKHO1 recipient mouse.

To address this question, we followed recipient mice up to 12 months after transplant (Figure 4A). We performed these experiments using both *Cdkn2a*-WT and *Cdkn2a*-KO GFP^+^ Hoxb8-FL cells. Transduced GFP^+^dTom^+^ Hoxb8-FL cells of all genotypes showed short-term (up to 6 weeks after transplant) contribution to peripheral blood leukocytes, as expected (40) (Figure 4B). Strikingly, some recipient mice later showed a rapid rise in GFP^+^dTom^+^ contribution to peripheral blood leukocytes, usually expressing the myeloid marker CD11b, which developed into a lethal acute leukemia (Figure 4, B and C). Leukemias were oligoclonal, and surface marker expression was variable (Supplemental Figure 4A), as expected from transformation of a pool of undifferentiated progenitor cells. While some leukemia clones showed reduced dTom expression levels, expression of GFP confirmed their derivation from transplanted Hoxb8-FL cells. Hoxb8-FL cell–derived leukemia was almost fully penetrant in *Cdkn2a*-WT MYB::PLEKHO1, *Cdkn2a*-KO MYB-TR, and *Cdkn2a*-KO MYB::PLEKHO1 recipient mice but was rarely observed in other genotypes and only with significantly longer latency (Figure 4D).

MYB-TR and MYB::PLEKHO1 leukemias displayed a surface marker profile consistent with a myeloid or myeloid-dendritic progenitor origin, with consistent expression of CD11b and variable expression of CD11c (dendritic marker), BST2 (pDC marker), CD117 (stem/progenitor marker), and CD115 and CX3CR1 (myeloid-dendritic progenitor markers) (44) (Figure 4E and Supplemental Figure 4A). Leukemias were negative for CD3 (T cell marker) and, except for rare B cell leukemia clones, negative for SCA1 (stem/progenitor marker), and CD19 and B220 (B cell markers) (Figure 4E and Supplemental Figure 4A).

Leukemias were transplantable, with a latency of 2–5 weeks in secondary recipient mice (Supplemental Figure 4B). Western blots confirmed expression of MYB-TR and MYB::PLEKHO1 in bone marrow cells from leukemic mice (Supplemental Figure 4C). *Cdkn2a*-WT MYB::PLEKHO1 leukemias showed intact expression of p16INK4A and p19ARF, while *Cdkn2a*-KO leukemias did not, confirming that MYB::PLEKHO1 is indeed able to induce leukemia independently of *Cdkn2a* loss (Supplemental Figure 4C).

These data show that BPDCN MYB fusions are able to induce leukemia in hematopoietic progenitor cells, while overexpression of MYB-WT is not. The fusion partner evidently plays a role in the leukemogenicity of the MYB fusion, since MYB::PLEKHO1 can induce leukemia independently while MYB-TR requires KO of *Cdkn2a*.

### MYB::PLEKHO1 can independently overcome G1/S arrest to induce leukemia.

To further characterize BPDCN MYB fusion–induced leukemias, we first performed transcriptomic analysis using RNA-Seq. For comparison, we used the Haemopedia dataset (45), which includes bulk RNA-Seq data from mouse hematopoietic stem, progenitor, and differentiated populations. We also included RNA-Seq data from WT Hoxb8-FL cells, either undifferentiated or differentiated in vitro to pDCs. Principal component analysis showed that, as expected, undifferentiated Hoxb8-FL cells were transcriptionally similar to lympho-myeloid progenitor populations, while Hoxb8-FL cell–derived in vitro–differentiated pDCs were similar to in vivo pDCs (Supplemental Figure 5A). Hoxb8-FL cell–derived MYB-TR and MYB::PLEKHO1-driven leukemias were positioned between myeloid progenitors, monocytes, and DCs (Supplemental Figure 5A), consistent with their surface protein marker expression (Supplemental Figure 4A).

**Figure 5:**
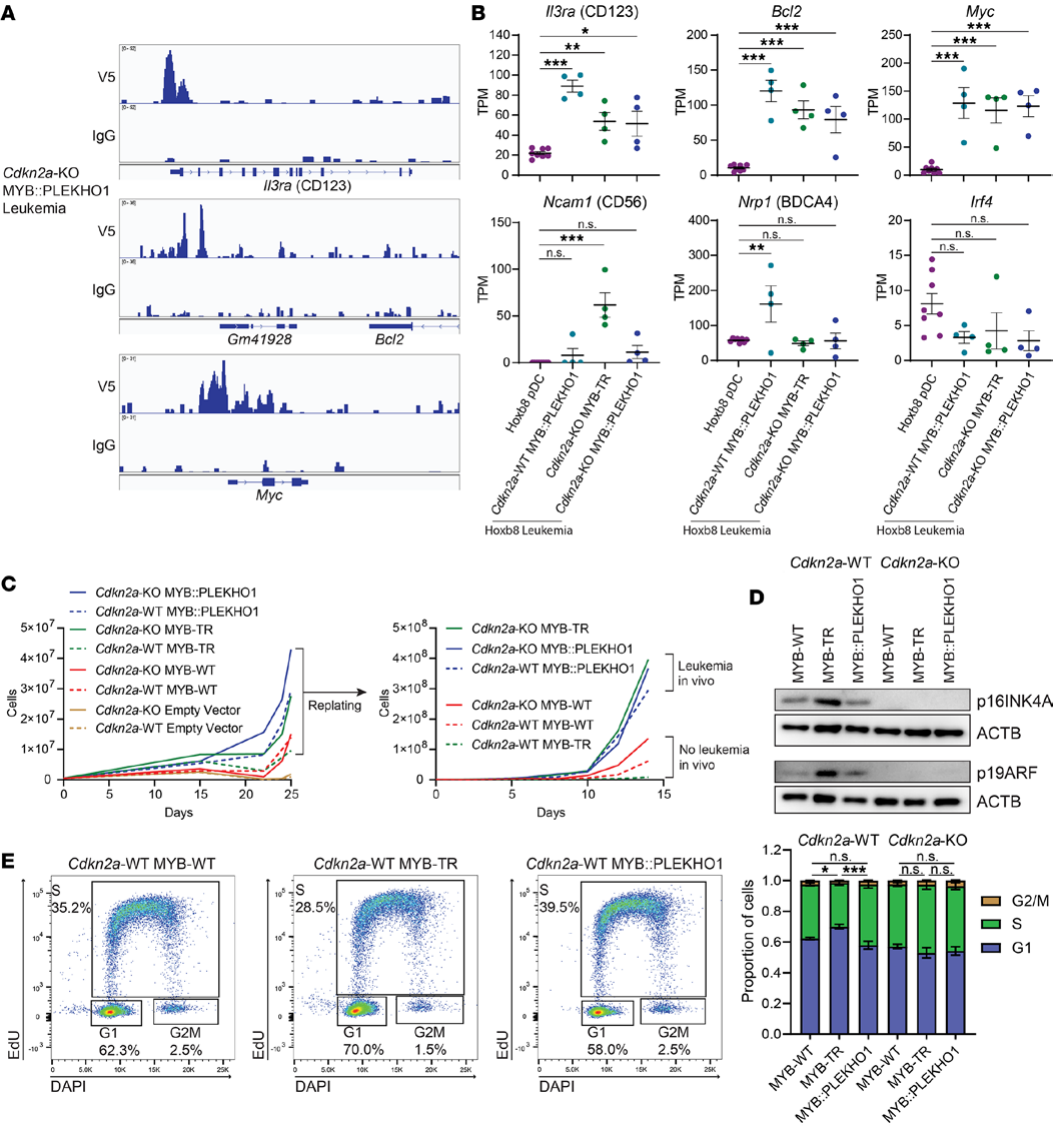
MYB::PLEKHO1 can independently overcome G1/S arrest to induce leukemia. (**A**) V5 and IgG CUT&RUN tracks at the *Il3ra*, *Bcl2*, and *Myc* loci in *Cdkn2a*-KO MYB::PLEKHO1 leukemia cells. (**B**) TPM of *Il3ra* (CD123), *Bcl2*, *Myc*, *Ncam1* (CD56), *Nrp1* (BDCA4), and *Irf4* in Hoxb8-FL cell–derived pDCs (*n* = 8) and Hoxb8-FL cell–derived leukemias of the indicated genotypes (*n* = 4 per genotype). (**C**) Growth curves of Hoxb8-FL.MS5 cells transduced with V5-MYB constructs of the indicated genotypes. (**D**) Western blots showing p16INK4A and p19ARF expression in Hoxb8-FL.MS5 cells of the indicated genotypes. (**E**) Cell cycle analysis of Hoxb8-FL.MS5 cells. (Left) Representative flow plots of the indicated genotypes. (Right) Quantification of cells in each cell cycle phase as a proportion of total cells (*n* = 4 per genotype). Significance tests compare proportion of cells in G1. (**A** and **E**) Data represent mean ± SEM. Significance determined by 1-way ANOVA with Tukey correction for multiple comparisons. *P <0.05, **P <0.01, ***P < 0.001.

To investigate how BPDCN MYB fusions induce leukemic transformation of Hoxb8-FL progenitor cells, we performed CUT&RUN in MYB::PLEKHO1 leukemia cells using an anti-V5 antibody to detect chromatin occupancy of the exogenously expressed fusion protein (Supplemental Table 4). This revealed binding of MYB::PLEKHO1 to the transcription start sites of BPDCN-associated genes such as *Il3ra* (CD123), *Bcl2*, and *Myc* (3) (Figure 5A), which were strongly expressed in leukemia cells (Figure 5B). BPDCN-associated genes such as *Ncam1* (CD56), *Nrp1* (BDCA4), and *Irf4* (46) were also expressed (Figure 5B), supporting the relevance of this mouse model to BPDCN. Genome-wide analysis revealed an enrichment of MYB::PLEKHO1 binding at transcription start sites of G2/M cell cycle–associated genes (Supplemental Figure 5B). MYB binding motifs were present at these binding sites, indicating binding of MYB::PLEKHO1 to DNA via the MYB DNA binding domain (Supplemental Figure 5, C and D). This confirms that BPDCN MYB fusions can initiate leukemia in hematopoietic progenitor cells associated with their binding to cell cycle genes.

To investigate why MYB::PLEKHO1 but not MYB-TR initiated leukemia in *Cdkn2a*-WT Hoxb8-FL cells, we developed a system to culture MYB-expressing Hoxb8-FL cells in vitro without requiring Hoxb8 activation. We cocultured Hoxb8-FL cells with the MS5 stromal cell line in media containing FLT3L but not estradiol (Hoxb8-FL.MS5 cells) and found that all genotypes of dTom^+^ MYB-expressing cells, but not dTom^–^ untransduced cells or dTom^+^ EV-transduced cells, were able to continue proliferating indefinitely (Figure 5C and Supplemental Figure 6A). The growth rate of MYB-expressing genotypes in this coculture system correlated with their leukemia-inducing potential in vivo (Figure 5C).

Western blot analysis showed increased p16INK4A and p19ARF in *Cdkn2a*-WT cells expressing MYB-TR compared with either MYB-WT or MYB::PLEKHO1 (Figure 5D and Supplemental Figure 6B). This suggests that MYB-TR activates the G1/S cell cycle checkpoint and could explain why MYB-TR Hoxb8-FL.MS5 cells have a slow growth rate in vitro, and MYB-TR is unable to induce leukemia in vivo without KO of *Cdkn2a*. This was supported by cell cycle analysis, which revealed an increased proportion of cells in G1 phase in *Cdkn2a*-WT MYB-TR cells compared with either *Cdkn2a*-WT MYB-WT or *Cdkn2a*-WT MYB::PLEKHO1 cells (Figure 5E). This was not the case in *Cdkn2a*-KO cells, where p16INK4A and p19ARF are not expressed (Figure 5, D and E). These data support the hypothesis that, in contrast to MYB-TR, the MYB::PLEKHO1 fusion may be sufficient to induce leukemia as a single event because it bypasses the G1/S checkpoint and does not induce strong expression of p16INK4A and p19ARF.

We performed RNA-Seq and CUT&RUN for V5 in Hoxb8-FL.MS5 cells to determine how MYB fusions initiate leukemia in these cells (Supplemental Tables 4 and 5). Comparing *Cdkn2a*-WT MYB-WT cells (which do not induce leukemia in vivo) with *Cdkn2a*-WT MYB::PLEKHO1 cells (which do induce leukemia), genes that showed both increased expression in MYB::PLEKHO1 cells and binding of MYB at the promoter were enriched for G2/M cell cycle regulators such as *Cdc20*, *Ccp110*, and *Kif20a* (Supplemental Figure 6, C–E). Together, these data support a model in which MYB fusions contribute to transformation by activating the G2/M transition and in which MYB::PLEKHO1 is sufficient to cause BPDCN on its own because it can uniquely activate G2/M while simultaneously facilitating bypass of the G1/S cell cycle checkpoint.

### BPDCN cells undergo loss of MYB and cell death upon ATRA treatment.

Given that the RARA ligand ATRA induces loss of MYB combined with cell death in ACC cells (31) and given that, like BPDCN, ACC also features recurrent MYB fusions (7, 8), we wondered whether BPDCN would show sensitivity to ATRA treatment.

**Figure 6:**
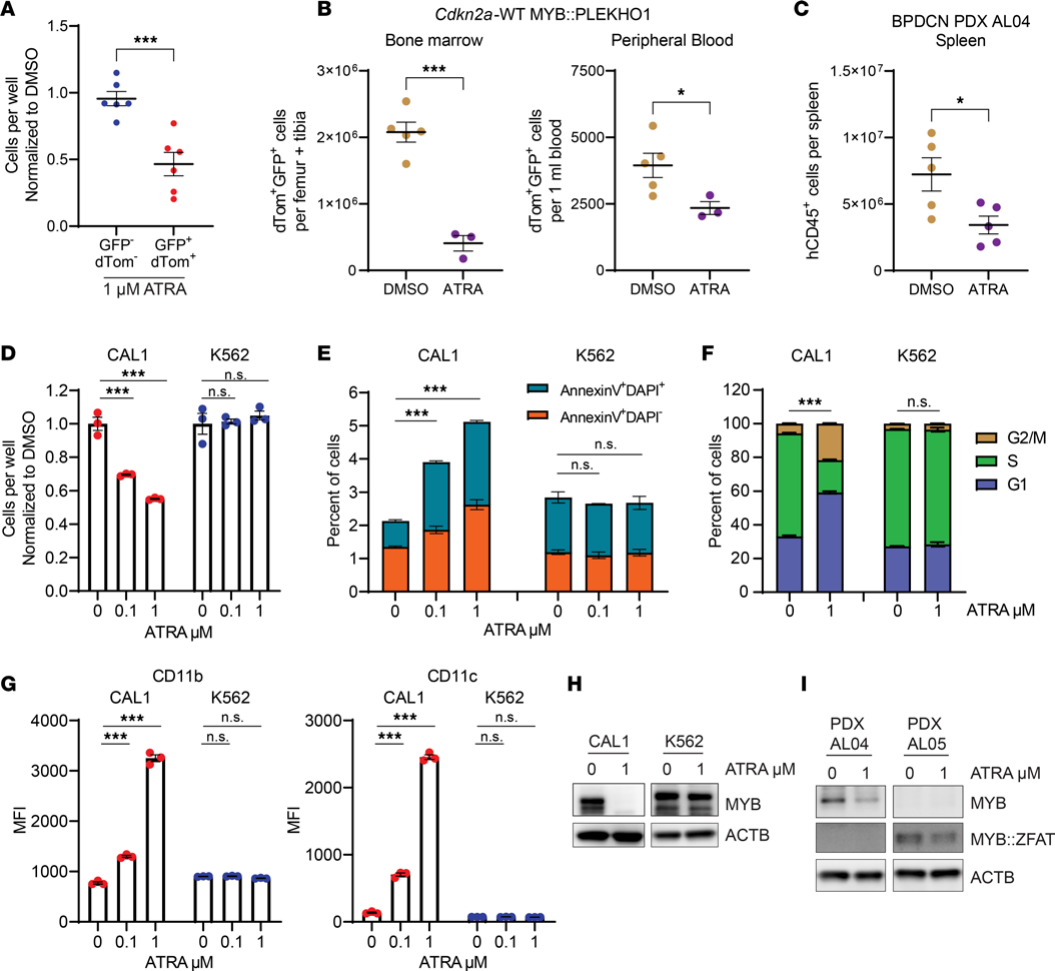
BPDCN cells undergo loss of MYB and cell death upon ATRA treatment. (**A**) Number of viable GFP^–^dTom^–^ and GFP^+^dTom^+^ spleen cells from leukemic *Cdkn2a*-KO MYB::PLEKHO1 recipient mice after 2 days of 1 μM ATRA treatment, normalized to 0 μM ATRA (*n* = 3 from each of 6 recipient mice). (**B**) Number of viable GFP^+^dTom^+^ cells per femur and tibia, and per 1 mL of peripheral blood of *Cdkn2a*-WT MYB::PLEKHO1 secondary recipient mice, treated daily with 20–40 mg/kg ATRA for 12 days (DMSO *n* = 5, ATRA *n* = 3). (**C**) Number of viable human CD45^+^ cells per spleen of BPDCN PDX AL04 recipient NSG mice treated daily with 20 mg/kg ATRA for 28 days (DMSO, *n* = 5; ATRA, *n* = 5). (**D**) Number of viable CAL1 and K562 cells after 2 days of ATRA treatment at the indicated dose, normalized to 0 μM ATRA (*n* = 3 per condition). (**E**) Annexin V apoptosis analysis of CAL1 and K562 cells after 2 days of ATRA treatment at the indicated dose (*n* = 3 per condition). Significance tests compare proportion of apoptotic cells (annexin V^+^DAPI^–^). (**F**) Cell cycle analysis of CAL1 and K562 cells after 2 days of ATRA treatment at the indicated dose (*n* = 3 per condition). Significance tests compare proportion of cells in G1. (**G**) Median fluorescence intensity for CD11b APC and CD11c APC-Cy7 in CAL1 and K562 cells after 2 days of ATRA treatment at the indicated dose (*n* = 3 per condition). (**H**) Western blots showing MYB expression in CAL1 and K562 after 2 days of ATRA treatment at the indicated dose. (**I**) Western blots showing MYB and MYB::ZFAT expression in BPDCN PDX AL04 and AL05 cells after 2 days of ATRA treatment at the indicated dose. (**A**–**G**) Data represent mean ± SEM. Significance determined by 2-tailed *t* test (**A**–**C**, and **F**) or 1-way ANOVA with Tukey correction for multiple comparisons (**D**, **E**, and **G**). *P <0.05, ***P <0.001.

We first treated *Cdkn2a*-KO MYB::PLEKHO1 leukemia cells harvested from mouse spleens with ATRA in vitro. dTom^+^GFP^+^ leukemia cells showed higher sensitivity to ATRA than the adjacent dTom^–^GFP^–^ endogenous normal splenocytes, indicating a specific effect on leukemic rather than normal hematopoietic cells (Figure 6A). In vivo ATRA treatment in secondary recipients of *Cdkn2a*-WT MYB::PLEKHO1 or *Cdkn2a*-KO MYB::PLEKHO1 leukemias resulted in a significant reduction in disease burden (Figure 6B and Supplemental Figure 7A). These data indicate that MYB-driven myeloid-dendritic cell leukemias are sensitive to ATRA, which was well tolerated at the doses used (Supplemental Figure 7B). We also found that ATRA treatment in vivo reduced the leukemic burden in NSG mice transplanted with a human BPDCN primary PDX (Figure 6C).

We next treated CAL1 (BPDCN) and K562 (AML) cells with ATRA, and we found that CAL1 was highly sensitive to ATRA while K562 was resistant (Figure 6D). This was accompanied by evidence of induction of apoptosis (Figure 6E), cell cycle arrest (Figure 6F), and expression of the differentiation markers CD11b and CD11c (Figure 6G and Supplemental Figure 7C). Like ACC cells, which are also highly sensitive to ATRA, CAL1 showed strongly reduced MYB protein upon ATRA treatment, which was not the case for K562 (Figure 6H and Supplemental Figure 7D). We also observed reduced MYB levels in 2 BPDCN PDX samples after ATRA treatment, one of which (AL05) carries the MYB::ZFAT fusion (Figure 6I and Supplemental Figure 7E).

These data provide evidence that BPDCN cells, with or without MYB fusions, may show sensitivity to ATRA treatment via loss of MYB, as in ACC.

## Discussion

Truncated forms of MYB were first discovered as viral inducers of avian leukemias and were subsequently shown to contribute to leukemic transformation in mouse hematopoietic cells (47), but whether and how they might be involved in human leukemia has remained unclear. Here we demonstrate that MYB fusions, present in around 20% of adult BPDCNs (3, 4) (higher in pediatric patients and the only detected mutation in some cases) (2) increase the binding of MYB to DNA specifically at G2/M cell cycle genes. MYB fusions, but not MYB-WT, are able to induce myeloid/dendritic leukemia in vivo when expressed in hematopoietic progenitor cells. We also use ATRA to target expression of MYB in BPDCN models.

We used CUT&RUN to profile DNA binding of MYB in pDCs and BPDCN. Regardless of whether MYB fusions were present, we observed a switch from binding at dendritic lineage genes in normal pDCs to binding at G2/M cell cycle genes in BPDCN. Binding at dendritic lineage genes occurred mainly at sites containing TCF4 DNA binding motifs, the master transcriptional regulator of the pDC lineage (35, 36), while binding at cell cycle genes occurred at MYB motifs. This indicates a switch in the function of MYB during BPDCN leukemogenesis, from cooperative regulator of dendritic lineage genes in normal pDCs to direct regulator of cell cycle genes in BPDCN.

MYB binding motifs are present at G2/M genes owing to the involvement of B-MYB in their normal regulation during the cell cycle (37, 48). A role for MYB in direct control of the cell cycle via aberrant binding to B-MYB binding sites is possible given that MYB can compensate for loss of B-MYB in activation of Cyclin B1 expression in AML cells (49). We suggest that, in BPDCN, and potentially in other cancers, MYB contributes to cell cycle dysregulation by binding to and activating G2/M cell cycle genes using DNA motifs normally targeted by B-MYB.

Our K562 MYB-V5–knock-in experiments, in which MYB-WT, MYB-TR, or MYB::PLEKHO1 were expressed at physiological levels from the endogenous MYB promoter, strongly argue that MYB fusions intrinsically bind to DNA with higher affinity than MYB-WT, specifically at G2/M cell cycle genes. Our proposed model for the oncogenicity of MYB fusions in BPDCN is that they directly induce aberrant binding to G2/M cell cycle promoters via B-MYB binding sites, which results in deregulated expression of these genes and overriding of the G2/M checkpoint.

In the small cohort of patients reported by Suzuki et al. (2), MYB fusions were identified in all 5 pediatric BPDCNs, without other cancer driver mutations. This raised the possibility that MYB fusions could be sufficient to initiate BPDCN in some contexts. Our findings confirm that, in a mouse model, expression of MYB::PLEKHO1 is sufficient to initiate leukemia in hematopoietic progenitor cells. A caveat to these findings is that we cannot rule out that residual effects of the Hoxb8-ER transgene contribute to survival of these leukemia cells in vivo or that Hoxb8-FL cells may represent a nonphysiological hematopoietic progenitor state. However, the marked differences in leukemogenicity between our MYB constructs strongly suggest that MYB fusions identified in patients with BPDCN do indeed intrinsically possess leukemia initiating potential.

In a previous study, overexpression of MYB-WT in the entire hematopoietic system was sufficient to induce lymphoid or myeloid leukemias in mice (50). Conversely, in our Hoxb8-FL system, MYB-WT caused impaired differentiation of hematopoietic progenitor cells but did not induce leukemia, while BPDCN-associated MYB fusions were able to initiate leukemia. It is possible that overexpression of MYB-WT can induce leukemic transformation in hematopoietic stem cells, while the stronger activation of cell cycle genes by MYB fusions is more capable of transforming hematopoietic progenitor cells that have lost intrinsic self-renewal capacity. Interestingly, we found that, in the case of MYB-TR (which models the MYB::ZFAT fusion recurrent in BPDCN) (2), overriding the G1/S checkpoint by *Cdkn2a* KO was also required for leukemic transformation. This is consistent with the preponderance of *CDKN2A* deletions in patients with BPDCN (22, 24–26), which may indicate a general requirement to reactivate cell cycling to transform an otherwise quiescent terminally differentiated pDC or pDC progenitor (17).

In contrast to MYB-TR, MYB::PLEKHO1 did not require *Cdkn2a* KO to initiate leukemia in Hoxb8-FL cells. We observed higher p16INK4A and p19ARF expression in MYB-TR–expressing cells than in MYB-WT– or MYB::PLEKHO1-expressing cells. Since these proteins inhibit the transition from G1 to S phase, this could explain why MYB-TR–expressing cells did not independently undergo transformation. KO of *Cdkn2a* abolishes expression of p16INK4A and p19ARF, allowing cell cycle progression and leukemic transformation. Determining why MYB::PLEKHO1 does not induce *Cdkn2a* expression to the same extent as MYB-TR is a topic for future research. Nonetheless, together, our findings support a 2-hit model for BPDCN oncogenesis, in which both the G1/S and G2/M transitions must be activated to initiate the disease (Supplemental Figure 7F).

The retinoic acid pathway has multiple cellular functions including differentiation, apoptosis, and cell cycle control (51). In the hematopoietic system, ATRA has been shown to influence hematopoietic stem cell dormancy (52), and retinoic acid receptors have been suggested to directly oppose the function of MYB in hematopoietic self-renewal and differentiation (53, 54). In ACC — a solid tissue cancer, which, like BPDCN, shows recurrent MYB fusions — ATRA reduces MYB expression and inhibits tumor growth (7, 8, 31). We found that our BPDCN MYB fusion–induced mouse leukemias showed sensitivity to ATRA in vitro and in vivo, while CAL1 human BPDCN cells displayed differentiation, cell cycle arrest, and apoptosis upon ATRA treatment in vitro. ATRA treatment caused loss of MYB in CAL1, as well as in BPDCN PDX samples, which also showed reduced leukemia burden in recipient mice after ATRA treatment in vivo. Unlike in APL, which is driven by the PML::RARA fusion (30), ATRA should not necessarily be expected to show efficacy as a monotherapy in BPDCN. However, together our findings substantiate the concept of opposing roles of MYB and the retinoic acid pathway in hematopoietic cells and support further research into ATRA in the context of BPDCN.

## Methods

### Sex as a biological variable.

Sex was not considered as a biological variable.

### CUT&RUN.

Normal pDCs were obtained from healthy donor peripheral blood samples purified by Ficoll-Paque (Cytiva, 17144003). Non-pDCs were depleted using magnetic bead separation (Miltenyi Biotec, 130-097-415), and CD45^+^CD123^+^CD4^+^BDCA2^+^ pDCs were FACS purified using the following antibodies: CD45 FITC (BioLegend, 368508), CD123 APC (BD Biosciences, 658172), CD4 PE (BioLegend, 344606), and BDCA2 PE-Cy7 (BioLegend, 354213). Primary BPDCN cells were obtained from patient bone marrow samples by FACS-purifying CD45^lo^CD123^+^CD4^+^BDCA2^+^ BPDCN cells using the same antibodies. BPDCN PDX cells were obtained by depleting mouse cells from recipient spleen samples using magnetic bead separation (Miltenyi Biotec, 130-104-694). Mouse GFP^+^dTom^+^ leukemia cells were FACS purified from recipient spleen samples.

CUT&RUN was performed essentially as described (32) using 100,000 cells per sample. Cell membranes were permeabilized using 0.02% digitonin. Antibodies were used at 1/100 dilution with overnight binding at 4°C. pAG-MNase was purchased from Epicypher (catalog 15-1016) or Cell Signaling Technology (catalog 40366) and used at the recommended dilution. Binding of pAG-MNase was performed for 1 hour at 4°C and MNase digestion was performed for 30 minutes at 0°C. DNA was extracted by organic extraction as described (32). Antibodies used were: H3K27ac (Cell Signaling Technology, 8173), MYB (Abcam, ab45150), IgG (Cell Signaling Technology, 2729), V5 (Thermo Fisher Scientific, R960-25; K562 cells), V5 (Cell Signaling Technology, 13202; K562 cells), V5 (Abcam, ab15828; Hoxb8-FL cells and mouse leukemia cells).

CUT&RUN library prep was performed using the NEBNext Ultra II DNA Library Prep Kit largely as described (38) using AMPure XP (Beckman Coulter, A63880) after PCR size selection (0.4× negative selection to remove large DNA molecules followed by 1.2× positive selection to retain nucleosome and transcription factor-bound DNA fragments). PCR amplification was performed using 14 cycles of 10 seconds annealing/extension. DNA fragments were sequenced using 35 bp paired-end sequencing.

CUT&RUN reads were aligned to the hg38 or mm10 genomes using Bowtie2 (RRID:SCR_016368) and the following parameters: --very-sensitive-local --soft-clipped-unmapped-tlen --no-unal --no-mixed --no-discordant --dovetail --phred33 -I 10 -X 700. Binding sites were called using MACS2 (RRID:SCR_013291) and the following parameters: -q 0.01 --keep-dup all. Footprinting analysis was performed as described (38, 55). Binding sites were annotated using HOMER (RRID:SCR_010881). Motifs were called using HOMER, with filtering for target regions mapping to repetitive sequence.

Differential binding analysis was performed using DiffBind (RRID:SCR_012918), with normalization performed using the RLE method and analysis using DESeq2. For pDC versus BPDCN PDX differential binding of MYB, number of reads within binding peaks were used as normalization factors. For K562 differential binding of MYB, the number of reads mapping to the *E*. *coli* K12 genome were used as spike-in normalization factors (56).

Gene list overlaps were identified using MSigDB (RRID:SCR_016863). Gene lists were overlapped against the CP:Reactome gene sets. Gene set enrichment analysis was performed using GSEA software (RRID:SCR_003199) and published RNA-Seq data (20).

### Virus production and transduction.

Retro- and lentivirus were produced using 293T cells and standard procedures. 293T cells growing in 10 cm dishes were transfected with 60 μL Lipofectamine 2000 (Thermo Fisher Scientific, 11668500) and, for lentivirus: 10.8 μg psPAX2 (Addgene, 12260), 2.4 μg VSV.G (Addgene, 12259), and 10.8 μg expression plasmid, or for retrovirus: 12 μg pCL-Eco (Addgene, 12371) and 12 μg expression plasmid. Media were changed at 24 hours after transfection, and the virus was harvested at 48 hour and 72 hours. Target cells were transduced using 4 μg/mL polybrene (Santa Cruz Biotechnology Inc., sc-134220) and 90 minutes at 1,000*g* spinfection. Transduced cells were resuspended in fresh media 24 hours after transduction.

### MYB-V5 knock-in.

MYB-V5–knock-in K562 cells were generated using a modified pFETCH vector (Addgene, 63934) essentially as described (57). K562 cells constitutively expressing Cas9 were first generated using the lentiCRISPRv2 vector (58). These cells were cotransfected with sgRNAs targeting either the ENST00000367814.8 *MYB* stop codon (ACATTTCCAGAAAAGCATTA and TGAAAACCATAATGCTTTTC) or end of exon 9 (TCTGTAAGTAGAATTGTGAA), and the pFETCH construct containing a V5 tag, P2A site, and Neomycin resistance gene, with homology arms targeting either exon 15 (MYB-WT) or exon 9 (MYB-TR and MYB::PLEKHO1). The MYB::PLEKHO1 vector also contained the exon 6 (final exon) coding sequence of ENST00000369124.5 *PLEKHO1* before the V5 tag. Successfully targeted cells expressed MYB-WT, MYB-TR, or MYB::PLEKHO1 with in-frame knock-in of V5, P2A, and NeoR and were selected for using 200 μg/mL G418 (Thermo Fisher Scientific, MT61234RG).

### Western blots.

Protein lysates from K562, CAL1, and PDX cells were prepared using RIPA buffer (Thermo Fisher Scientific, 89900). Protein concentrations were measured using BCA (Thermo Fisher Scientific, 23227), and 5 μg protein was loaded per well. Protein lysates from Hoxb8-FL cells and mouse bone marrow were prepared using direct lysis (59). Cells were resuspended in SDS-PAGE sample buffer (Thermo Fisher Scientific, NP0007) with 5% 2-mercaptoethanol (MilliporeSigma, M3148). Lysates were frozen on dry ice and were then denatured by heating at 99°C for 10 minutes with rapid shaking, and the equivalent of 10,000 cells was loaded per well. Antibodies used for Western blots were: MYB (Abcam, ab45150), V5 (Cell Signaling Technology, 13202), ACTB (Santa Cruz Biotechnology Inc., sc-47778), GAPDH (Cell Signaling Technology, 2118), p16INK4A (Cell Signaling Technology, 29271), and p19ARF (Cell Signaling Technology, 77184). Protein band quantification was performed using ImageJ (NIH; RRID:SCR_003070).

### Generation of Hoxb8-FL cells.

Hoxb8-FL cells were generated as described (40) from mice constitutively expressing Cas9 and GFP from the Rosa26 locus (41). Unfractionated bone marrow cells were cultured for 2 days in RPMI-1640 (Thermo Fisher Scientific, 11875119) + 10% FBS (MilliporeSigma, F2442) + 1% penicillin-streptomycin (Pen/Strep; Thermo Fisher Scientific, 15140122) supplemented with 10 ng/mL mIL-3 (BioLegend, 575504), 10 ng/mL mIL-6 (BioLegend, 575704), and 25 ng/mL mSCF (BioLegend, 579706) before retroviral transduction of the HA-ER-Hoxb8 vector (60) (provided by the David Sykes lab; Massachusetts General Hospital, Boston, Massachusetts, USA). Transduced cells were cultured in RPMI-1640 + 10% FBS + 1% Pen/Strep supplemented with 1 μM estradiol (MilliporeSigma, E2758) and 50 ng/mL mFLT3L (BioLegend, 550706).

### V5-MYB expression in Hoxb8-FL cells.

V5-tagged mouse MYB was expressed in Hoxb8-FL cells by lentiviral transduction using the pRRL.SFFV.IRES.dTom vector (provided by the Benjamin Ebert lab; Dana-Farber Cancer Institute, Boston, Massachusetts, USA). MYB-WT was the full ENSMUST00000020158.8 *Myb* coding sequence, MYB-TR was exons 1–9 only, and MYB::PLEKHO1 was exons 1–9 of *Myb* followed by the exon 6 (final exon) coding sequence of human ENST00000369124.5 *PLEKHO1*.

For in vitro differentiation, 3 days after transduction of V5-MYB, Hoxb8-FL cells were washed in RPMI-1640 + 10% FBS + 1% Pen/Strep and were then resuspended in the same media supplemented with 50 ng/mL mFLT3L and cultured for 7 days. Cells were resuspended in fresh media on day 2 and day 4. On day 7, cells were stained for flow cytometry with the following antibodies: CD11b AF700 (BioLegend, 101222), CD11c PE-Cy7 (BioLegend, 117317), and B220 APC-Cy7 (BioLegend, 103224).

For in vivo experiments, 3 days after transduction of V5-MYB, Hoxb8-FL cells were transplanted into lethally irradiated (2 × 5 Gy) C57BL/6J recipient mice (The Jackson Laboratory, 000664) by i.v. injection with 250,000 support bone marrow cells. Numbers of dTom^+^ cells transplanted per recipient: *Cdkn2a*-WT 235,000–825,000; *Cdkn2a*-KO Empty 235,000–4,200,000; *Cdkn2a*-KO MYB-WT 235,000–825,000; *Cdkn2a*-KO MYB-TR 235,000–2,200,000; *Cdkn2a*-KO MYB::PLEKHO1 190,000–1,575,000. For secondary transplants, 1.65–2.75 million unfractionated bone marrow cells were transplanted into sublethally irradiated (2 × 2.5 Gy) recipient mice.

For in vivo differentiation experiments, bone marrow and spleen cells were harvested from recipient mice 7 days after transplantation, treated with Fc block (BD Biosciences, 553141) and stained for flow cytometry with the following antibodies: BST2 APC (BioLegend, 127021), Siglec-H PerCP-Cy5.5 (BioLegend, 129613), CD19 BV605 (BioLegend, 115540), CD11c PE-Cy7, B220 APC-Cy7, and CD11b AF700.

For long-term in vivo experiments, serial peripheral blood samples were obtained from the tail vein of recipient mice, and leukocytes were stained for flow cytometry with the following antibodies: CD3 APC (BioLegend, 100235), CD11c PE-Cy7, B220 APC-Cy7, CD19 BV605, and CD11b AF700. Recipient mice were euthanized when showing signs of malignancy. Mice with greater than 20% GFP^+^dTom^–/+^ Hoxb8-FL-derived cells in either the bone marrow or spleen were classified as leukemic.

For MS5 coculture, 3 days after transduction of V5-MYB, Hoxb8-FL cells were resuspended in RPMI-1640 + 10% FBS + 1% Pen/Strep supplemented with 50 ng/mL mFLT3L and cultured in dishes preplated with MS5 murine bone marrow stromal cells (RRID:CVCL_2128). Media were refreshed every 2–3 days, and cells floating in suspension were used for analysis.

### RNA-Seq.

RNA was extracted from Hoxb8-FL cells and Hoxb8-FL cell–derived leukemias using RNeasy Mini Kit (Qiagen, 74104). Library prep was performed by the Molecular Biology Core Facility at Dana-Farber Cancer Institute using KAPA mRNA HyperPrep Kit (Roche, KK8580) and sequenced using 150 bp paired-end sequencing. Haemopedia data (45) were downloaded from Gene Expression Omnibus (GEO; accession no. GSE116177). Reticulocytes and in vitro–cultured samples were excluded.

Raw reads were trimmed using Trimmomatic (RRID:SCR_011848) with settings ILLUMINACLIP:TruSeq3-PE-2.fa:2:30:10:1:true MINLEN:20 and were then aligned to the mm10 genome using STAR (RRID:SCR_004463) and default settings. Duplicate reads were removed with Picard (RRID:SCR_006525), and reads were assigned to gene exons using featureCounts (RRID:SCR_012919). Nonexpressed genes were removed and RUVSeq (RRID:SCR_006263) was used for batch correction by normalizing to 25 genes uniformly expressed in mouse bone marrow (HRT Atlas; RRID:SCR_025119): *Cpsf3*, *Srp72*, *Nsd1*, *Zfp91*, *Plaa*, *Mapre1*, *Zmat2*, *Api5*, *Rad21*, *Smu1*, *Gtf2h1*, *Stt3b*, *Ddx17*, *Utp6*, *Sec24c*, *Canx*, *Caprin1*, *Atxn10*, *Bzw1*, *Sf3b2*, *Vps36*, *Sec31a*, *Copb1*, *Yeats4*, and *Psmc6*. PCA was performed on VST-transformed normalized counts using DESeq2 (RRID:SCR_015687). Differential expression analysis between Hoxb8-FL.MS5 cell genotypes was performed separately on raw counts using DESeq2.

### Cell cycle analysis.

Cell cycle analysis was performed essentially as described (61). Cells in culture were treated with 10 μM EdU (MedChem Express, HY-118411) for 1 hour before harvesting. Cells were washed in PBS and fixed by resuspension in PBS + 4% paraformaldehyde (Santa Cruz Biotechnology Inc., sc-281692) for 15 minutes at 22°C. Cells were washed in PBS + 2% FBS and permeabilized in 70% ethanol for 30 minutes on ice; they were then washed twice in cold PBS. Cells were resuspended in the Click reaction mixture for 30 minutes at 22°C: PBS + 1 mM CuSO_4_ (MilliporeSigma, C1297), 0.1 mM THPTA (Click Chemistry Tools, 1010), 100 mM sodium ascorbate (VWR, 95035), 2 μM AZDye 647 Azide (Click Chemistry Tools, 1299). Cells were washed twice in PBS + 2% FBS and then resuspended in PBS + 1 μg/mL DAPI (Cell Signaling Technology, 4083) + 0.1 μg/mL RNase A (Thermo Fisher Scientific, EN0531) and incubated for 1 hour at 22°C before flow cytometry analysis.

### ATRA treatment.

For in vitro ATRA treatment, cells were treated with the indicated doses of ATRA (MedChem Express, HY-14649) and 0.1% DMSO for 2 days. PDX cells from recipient spleens were cocultured with MS5 cells and 10 ng/mL hSCF (PeproTech, 300-07), hFLT3L (PeproTech, 300-19), hIL-3 (PeproTech, 200-03), and hIL-7 (PeproTech, 200-07) and were purified by bead-based mouse cell depletion (Miltenyi Biotec, 130-104-694) before lysis for Western blots.

For in vivo ATRA treatment of Hoxb8-FL cell–derived leukemias, 0.5–1.0 million unfractionated bone marrow cells from leukemic *Cdkn2a*-WT MYB::PLEKHO1 or *Cdkn2a*-KO MYB::PLEKHO1 primary recipient mice were transplanted into sublethally irradiated (2 × 2.5 Gy) secondary recipients. Starting 3 days after transplant, ATRA was administered by daily i.p. injections at a dose of 20–40 mg/kg for 12–21 days as indicated in figure legends. For in vivo ATRA treatment of BPDCN PDX, 0.7 million PDX cells were transplanted into sublethally irradiated (2 × 1 Gy) NSG recipient mice (The Jackson Laboratory, 005557). Starting after confirmation of engraftment by peripheral blood analysis, ATRA was administered by daily i.p. injections at 20 mg/kg for 28 days. ATRA was dissolved in DMSO and diluted 1/10 in 10% w/v 2-hydroxypropyl-β-cyclodextrin (MedChem Express, HY-101103) for administration. All mice were euthanized at the end of the treatment period.

For differentiation marker and annexin V analysis, CAL1 and K562 cells were treated with Fc block (BD Biosciences, 564219) before being stained for CD11b APC (BioLegend, 101212) and CD11c APC-Cy7 (BioLegend, 337218). Cells were then washed and resuspended in annexin V binding buffer (BioLegend, 422201) with annexin V FITC (BioLegend, 640906) for 15 minutes at 22°C. They were then diluted in binding buffer containing DAPI and fluorescent counting beads (Thermo Fisher Scientific, C36950) for flow cytometry analysis.

### Statistics.

Statistical tests were performed using GraphPad Prism 9 software. A 2-tailed *t* test was used to compare 2 groups, and 1-way ANOVA with Tukey correction for multiple comparisons was used to compare 3 or more groups. Data were summarized as mean ± SEM. Log-rank test was used to compare survival curves. *P* < 0.05 was considered significant.

### Study approval.

All studies were approved by Dana-Farber Cancer Institute IRBs and conformed to guidelines for ethical research conduct in the Declaration of Helsinki, and patients provided written informed consent. All animal experiments were performed with approval from the Dana-Farber Cancer Institute Animal Care and Use Committee.

## Author contributions

CAGB, JMB, KT, SC, and OA conducted experiments and analyzed data. HGR, KY, SR, EML, AK, ER, and JAD assisted in experimental design and data interpretation. JPB provided patient samples. CAGB and AAL designed the study, interpreted the data, and wrote the paper. All authors read and participated in editing the paper.

## Supplementary Material

Supplemental data

Unedited blot and gel images

Supplemental table 1

Supplemental table 2

Supplemental table 3

Supplemental table 4

Supplemental table 5

Supporting data values

## Version 1 11/05/2024

In-Press Preview

## Version 2 12/20/2024

Electronic publication
