# Supplementary material for: BPDCN MYB fusions regulate cell cycle genes, impair differentiation, and induce myeloid–dendritic cell leukemia
Source: JCI Insight. 2024 Dec 20;9(24):e183889. doi: 10.1172/jci.insight.183889 (PMC11665559; doi:10.1172/jci.insight.183889)
Supplement: Supplemental data [file jciinsight-9-183889-s128.pdf]

Figure S1

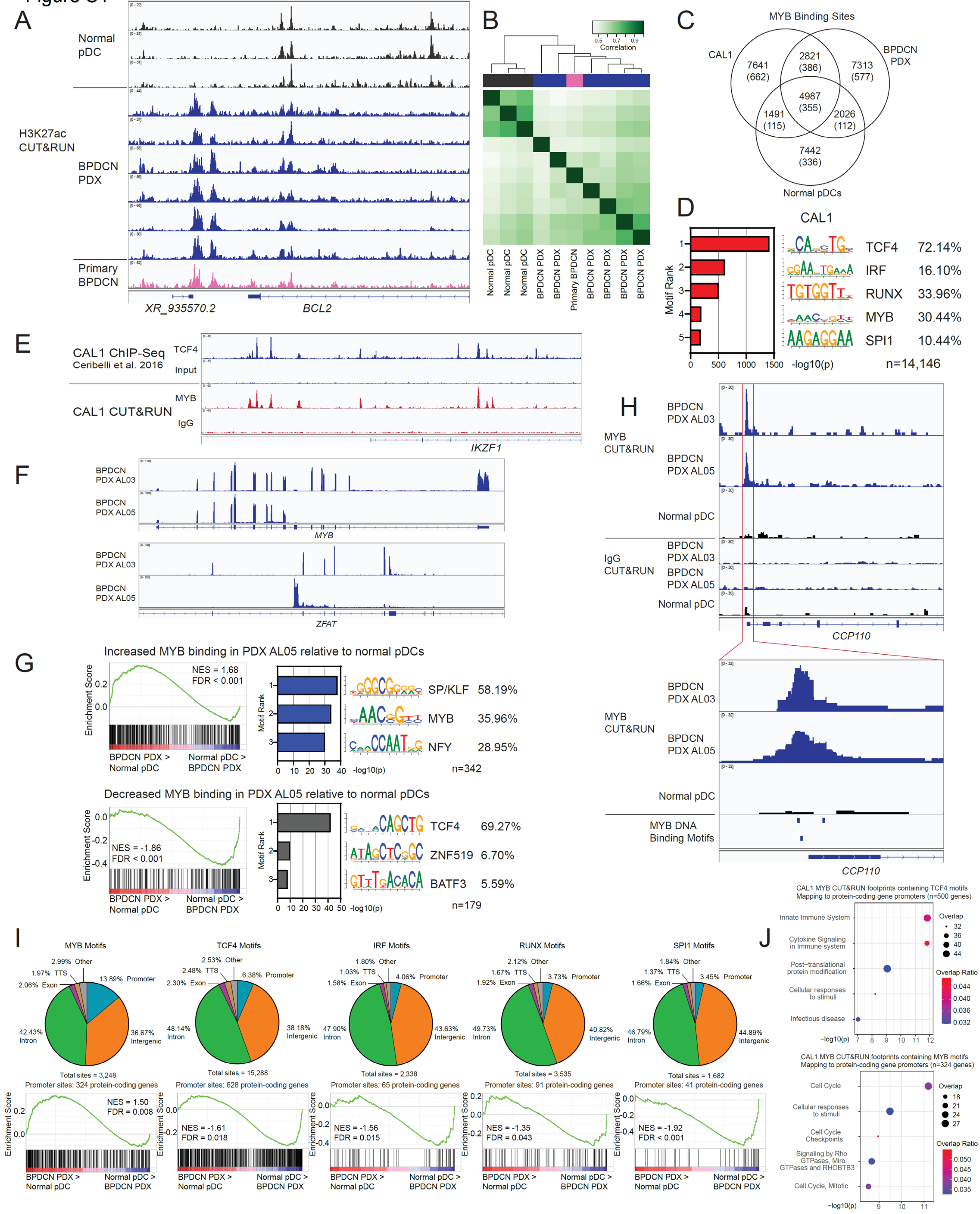

A
Normal pDC
H3K27ac CUT&RUN
BPDCN PDX
Primary BPDCN
XR_935570.2
BCL2
B
Correlation
0.5 0.7 0.9
Normal pDC
Normal pDC
Normal pDC
BPDCN PDX
BPDCN PDX
Primary BPDCN
BPDCN PDX
BPDCN PDX
BPDCN PDX
BPDCN PDX
C
MYB Binding Sites
CAL1
BPDCN PDX
Normal pDCs
7641 (662)
2821 (386)
7313 (577)
4987 (355)
1491 (115)
2026 (112)
7442 (336)
D
CAL1
Motif Rank
TCF4 72.14%
IRF 16.10%
RUNX 33.96%
MYB 30.44%
SPI1 10.44%
0 500 1000 1500 -log10(p)
n=14,146
E
CAL1 ChIP-Seq
Ceribelli et al. 2016
TCF4
Input
CAL1 CUT&RUN
MYB
IgG
IKZF1
F
BPDCN PDX AL03
BPDCN PDX AL05
MYB
BPDCN PDX AL03
BPDCN PDX AL05
ZFAT
G
Increased MYB binding in PDX AL05 relative to normal pDCs
Enrichment Score
NES = 1.68
FDR < 0.001
BPDCN PDX > Normal pDC
Normal pDC > BPDCN PDX
Motif Rank
SP/KLF 58.19%
MYB 35.96%
NFY 28.95%
0 10 20 30 40 -log10(p)
n=342
Decreased MYB binding in PDX AL05 relative to normal pDCs
NES = -1.86
FDR < 0.001
BPDCN PDX > Normal pDC
Normal pDC > BPDCN PDX
TCF4 69.27%
ZNF519 6.70%
BATF3 5.59%
0 10 20 30 40 50 -log10(p)
n=179
H
MYB CUT&RUN
BPDCN PDX AL03
BPDCN PDX AL05
Normal pDC
IgG CUT&RUN
BPDCN PDX AL03
BPDCN PDX AL05
Normal pDC
CCP110
MYB CUT&RUN
BPDCN PDX AL03
BPDCN PDX AL05
Normal pDC
MYB DNA Binding Motifs
CCP110
I
MYB Motifs
2.99% Other
1.97% TTS
2.06% Exon
13.89% Promoter
42.43% Intron
36.67% Intergenic
Total sites = 3,248
Promoter sites: 324 protein-coding genes
NES = 1.50
FDR = 0.008
TCF4 Motifs
2.53% Other
2.48% TTS
2.30% Exon
6.38% Promoter
48.14% Intron
38.18% Intergenic
Total sites = 15,288
Promoter sites: 628 protein-coding genes
NES = -1.61
FDR = 0.018
IRF Motifs
1.80% Other
1.03% TTS
1.58% Exon
4.06% Promoter
47.90% Intron
43.63% Intergenic
Total sites = 2,338
Promoter sites: 65 protein-coding genes
NES = -1.56
FDR = 0.015
RUNX Motifs
2.12% Other
1.67% TTS
1.92% Exon
3.73% Promoter
49.73% Intron
40.82% Intergenic
Total sites = 3,535
Promoter sites: 91 protein-coding genes
NES = -1.35
FDR = 0.043
SPI1 Motifs
1.84% Other
1.37% TTS
1.66% Exon
3.45% Promoter
46.79% Intron
44.89% Intergenic
Total sites = 1,682
Promoter sites: 41 protein-coding genes
NES = -1.92
FDR < 0.001
BPDCN PDX > Normal pDC
Normal pDC > BPDCN PDX
J
CAL1 MYB CUT&RUN footprints containing TCF4 motifs
Mapping to protein-coding gene promoters (n=500 genes)
Innate Immune System
Cytokine Signaling in Immune system
Post-translational protein modification
Cellular responses to stimuli
Infectious disease
-log10(p)
Overlap
Overlap Ratio
CAL1 MYB CUT&RUN footprints containing MYB motifs
Mapping to protein-coding gene promoters (n=324 genes)
Cell Cycle
Cellular responses to stimuli
Cell Cycle Checkpoints
Signaling by Rho GTPases, Miro GTPases and RHOBTB3
Cell Cycle, Mitotic

Figure S1: MYB aberrantly regulates G2/M cell cycle genes in BPDCN.

(A) H3K27ac CUT&RUN tracks at the *BCL2* locus in all samples from the indicated cell types (normal pDCs n=3, BPDCN PDX n=6, primary BPDCN n=1). (B) Clustering of H3K27ac CUT&RUN data from all samples from the indicated cell types (normal pDCs n=3, BPDCN PDX n=6, primary BPDCN n=1). Clustering was performed after counting of reads within pooled H3K27ac peaks and normalization to the total number of reads inside peaks for each sample. (C) Venn diagram showing number of MYB CUT&RUN peaks and, in brackets, number of peaks mapping to protein-coding gene promoters in the indicated cell types. (D) Top 5 ranked motifs enriched in MYB CUT&RUN peaks in CAL1 cells, and percent of peaks containing each motif. (E) TCF4 ChIP-seq (36) and MYB CUT&RUN tracks in CAL1 cells at the *IKZF1* locus. (F) Tracks showing RNA-sequencing read density at the *MYB* and *ZFAT* loci in AL03 and AL05 BPDCN PDX cells. (G) (Left) GSEA comparing normal pDCs and BPDCN PDX cells for genes showing differential MYB binding at promoter regions in BPDCN PDX AL05 relative to normal pDCs (n=346 genes increased and n=182 genes decreased MYB binding). (Right) Top 3 ranked motifs enriched in differentially bound MYB sites mapping to promoters, and percent of sites containing each motif. (H) MYB and IgG CUT&RUN tracks at the *CCP110* locus in the indicated cell types. (I) (Top) Locations of MYB CUT&RUN footprints containing the indicated motifs in CAL1 cells. (Bottom) GSEA comparing normal pDCs and BPDCN PDX cells for genes showing MYB CUT&RUN footprints containing the indicated motifs at promoter regions in CAL1 cells. (J) Top 5 ranked Reactome gene sets overlapping with genes showing MYB CUT&RUN footprints containing the indicated motifs at promoter regions in CAL1 cells.

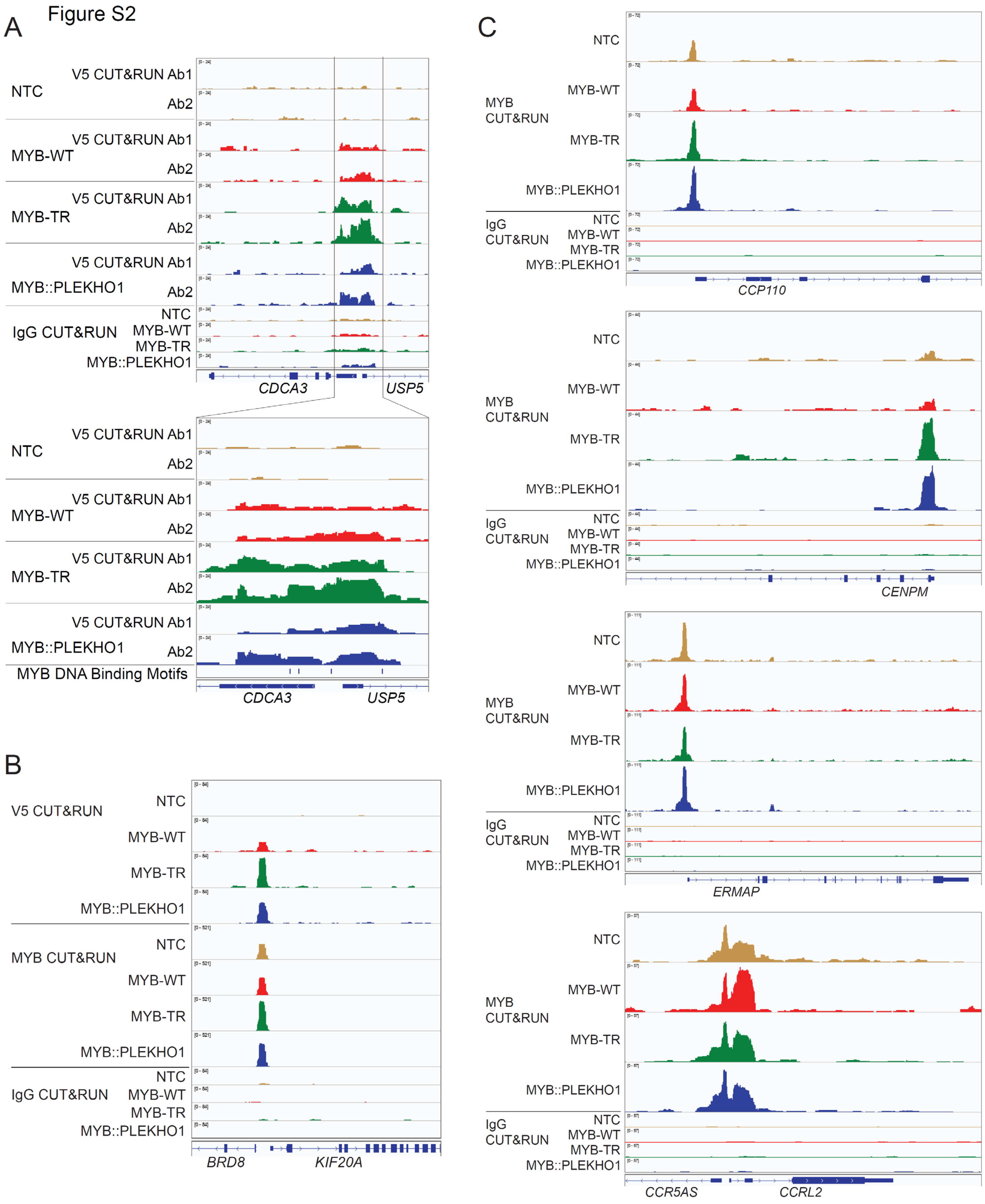

Figure S2: BPDCN MYB fusions cause increased binding to G2/M cell cycle genes.

(A) V5 and IgG CUT&RUN tracks at the *CDCA3/USP5* locus in MYB-V5 knock-in K562 cells of the indicated genotypes, for two different V5 antibodies. (B) V5, MYB and IgG CUT&RUN tracks at the *BRD8/KIF20A* locus in MYB-V5 knock-in K562 cells of the indicated genotypes. (C) MYB and IgG CUT&RUN tracks at the *CCP110*, *CENPM*, *ERMAP* and *CCRL2* loci in MYB-V5 knock-in K562 cells of the indicated genotypes.

Figure S3

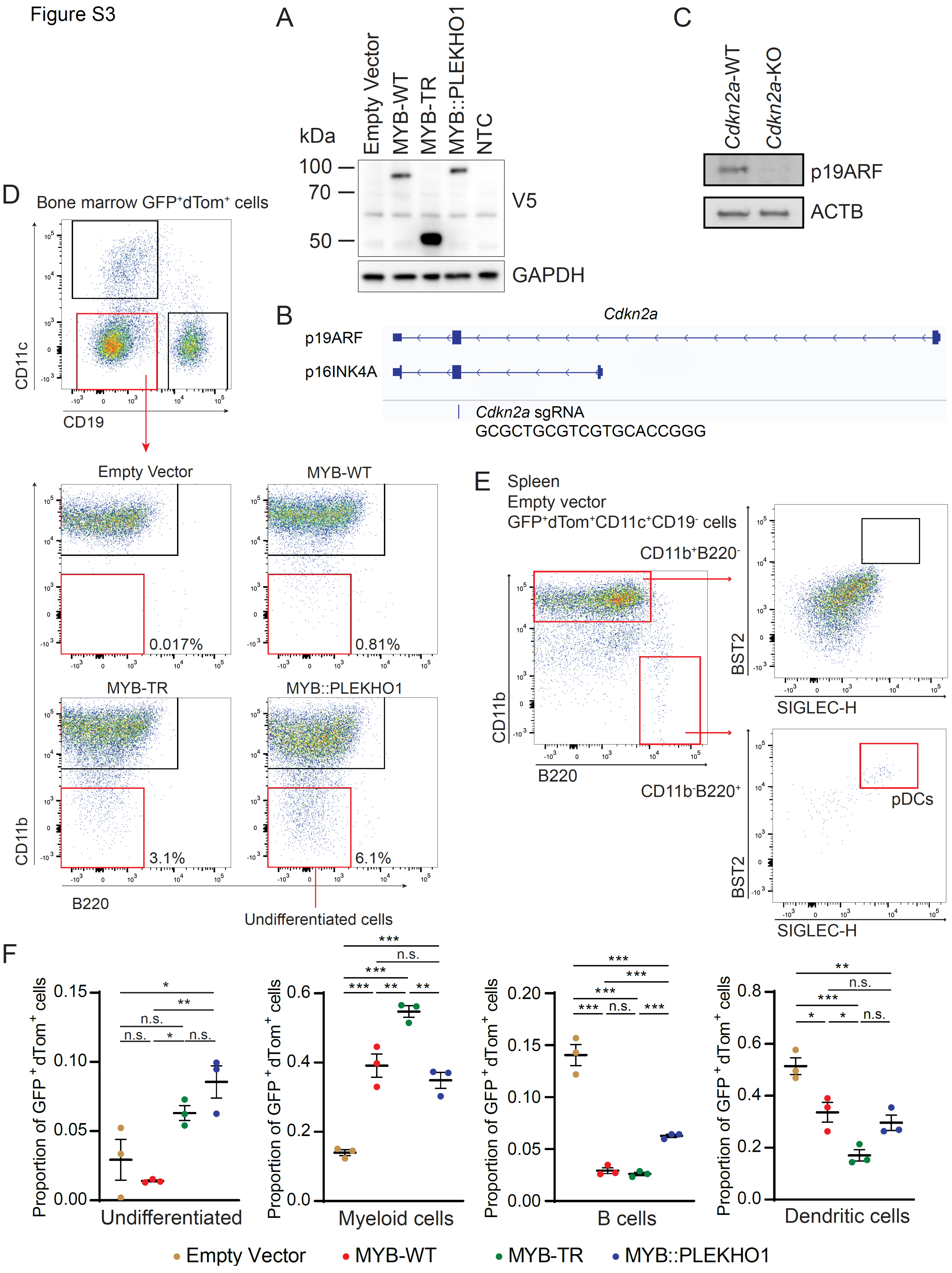

A
kDa
Empty Vector
MYB-WT
MYB-TR
MYB::PLEKHO1
NTC
100
70
50
V5
GAPDH
C
Cdkn2a-WT
Cdkn2a-KO
p19ARF
ACTB
B
Cdkn2a
p19ARF
p16INK4A
Cdkn2a sgRNA
GCGCTGCGTCGTGCACCGGG
D
Bone marrow GFP+dTom+ cells
CD11c
CD19
Empty Vector
MYB-WT
MYB-TR
MYB::PLEKHO1
0.017%
0.81%
3.1%
6.1%
CD11b
B220
Undifferentiated cells
E
Spleen
Empty vector
GFP+dTom+CD11c+CD19- cells
CD11b+B220-
CD11b-B220+
CD11b
B220
BST2
SIGLEC-H
pDCs
F
Proportion of GFP+dTom+ cells
Undifferentiated
Myeloid cells
B cells
Dendritic cells
Empty Vector
MYB-WT
MYB-TR
MYB::PLEKHO1

Figure S3: BPDCN MYB fusions impair differentiation in dendritic progenitor cells.

(A) Western blots showing expression of V5-MYB constructs in Hoxb8-FL cells. (B) Schematic showing mouse *Cdkn2a* locus p19ARF and p16INK4A open reading frames, with target location and sequence of *Cdkn2a*-KO sgRNA. (C) Western blot showing knockout of p19ARF in *Cdkn2a*-KO Hoxb8-FL cells. p16INK4A expression was too low to detect in undifferentiated Hoxb8-FL cells. (D) Representative flow cytometry plots of $GFP^{+}dTom^{+}$ bone marrow cells at day 7 of in vivo differentiation. Upper plot shows empty vector cells, lower plots show the indicated genotypes. (E) Representative flow cytometry plots of empty vector $GFP^{+}dTom^{+}CD11c^{+}CD19^{-}$ spleen cells at day 7 of in vivo differentiation. (F) Number of $GFP^{+}dTom^{+}$ spleen cells of the indicated cell types as a proportion of total $GFP^{+}dTom^{+}$ bone marrow cells (n=3 for each genotype). Undifferentiated $CD19^{-}CD11c^{-}CD11b^{-}B220^{-}$, myeloid cells $CD11c^{-}CD19^{-}CD11b^{+}$, B cells $CD11c^{-}CD19^{+}CD11b^{-}B220^{+}$, dendritic cells $CD11c^{+}CD19^{-}$. Data represent mean ± SEM. Significance determined by one-way ANOVA with Tukey correction for multiple comparisons. * $p<0.05$, ** $p<0.01$, *** $p<0.001$.

Figure S4

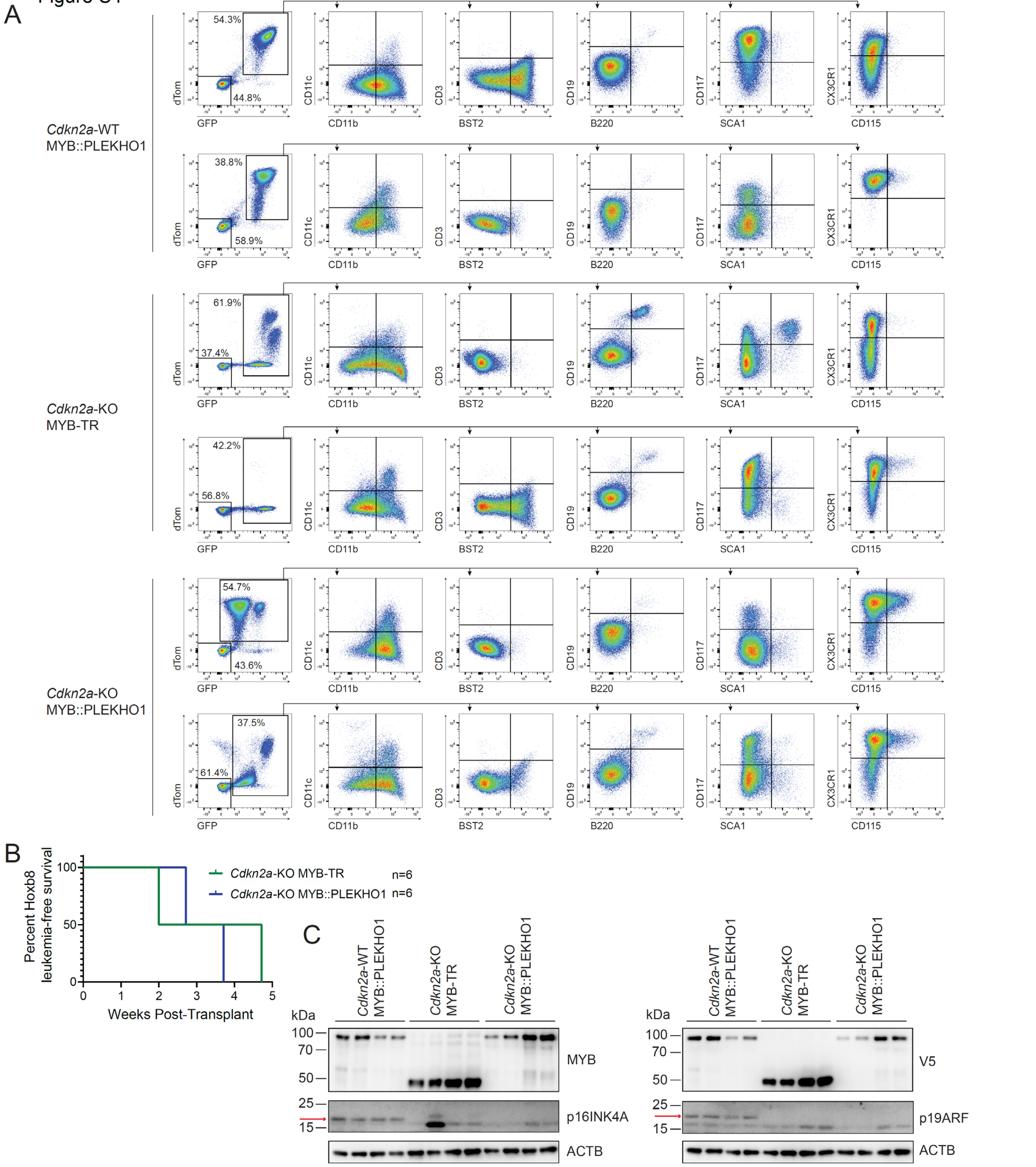

Figure S4: BPDCN MYB fusions expressed in hematopoietic progenitor cells induce myeloid-dendritic leukemia in vivo.

(A) Spleen flow cytometry plots for 6 leukemic Hoxb8-FL recipient mice of the indicated genotypes. (B) Hoxb8-FL cell-derived leukemia-free survival curves for secondary recipient mice of two primary *Cdkn2a*-KO MYB-TR and two primary *Cdkn2a*-KO MYB::PLEKHO1 leukemias (n=3 secondary recipients per primary leukemia). (C) Western blots showing expression of V5-MYB constructs, p16INK4A and p19ARF in unfractionated bone marrow cells from leukemic recipient mice of the indicated genotypes (n=4 per genotype).

Figure S5

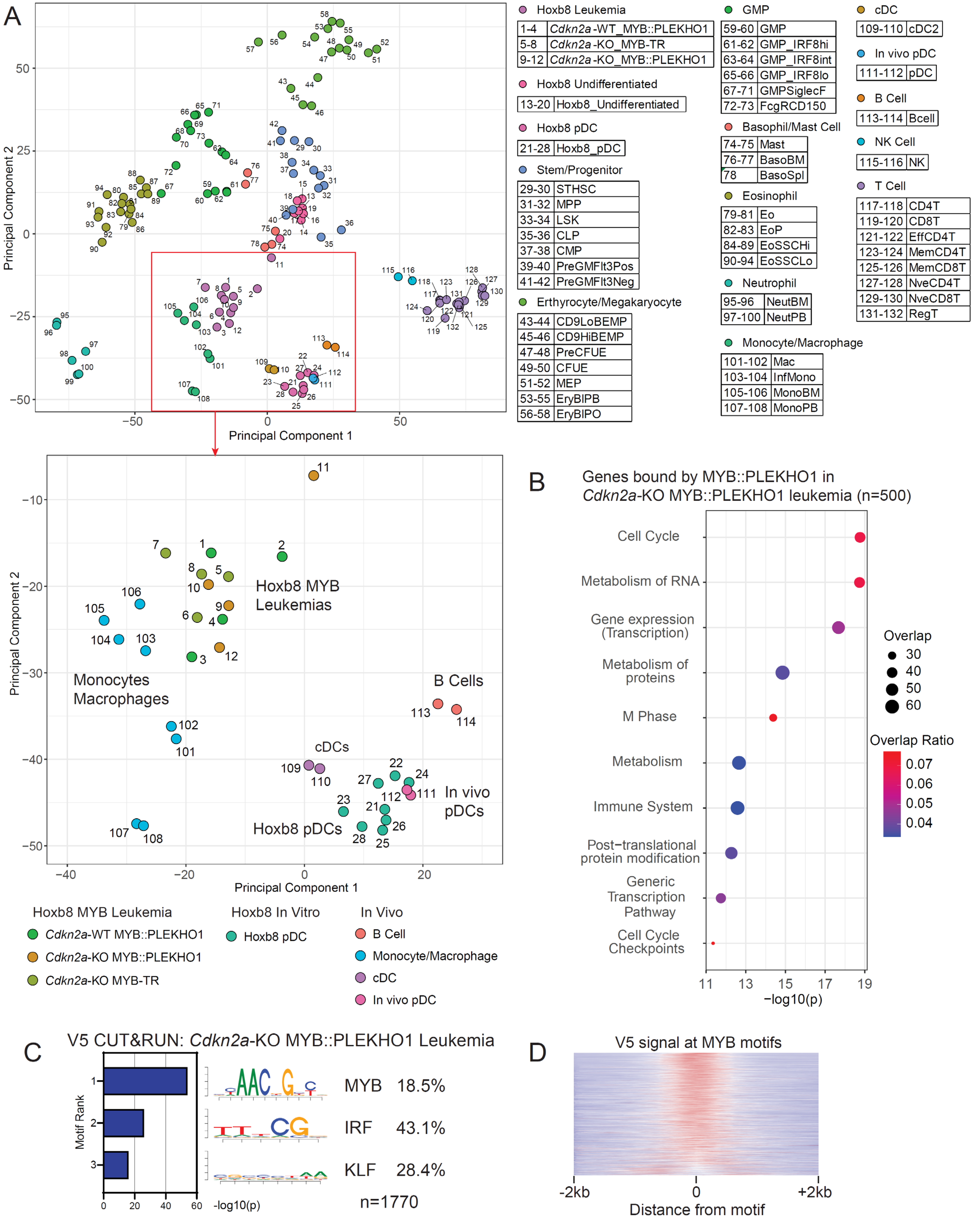

A
Principal Component 1
Principal Component 2
Hoxb8 Leukemia
1-4 Cdkn2a-WT_MYB::PLEKHO1
5-8 Cdkn2a-KO_MYB-TR
9-12 Cdkn2a-KO_MYB::PLEKHO1
Hoxb8 Undifferentiated
13-20 Hoxb8_Undifferentiated
Hoxb8 pDC
21-28 Hoxb8_pDC
Stem/Progenitor
29-30 STHSC
31-32 MPP
33-34 LSK
35-36 CLP
37-38 CMP
39-40 PreGMFlt3Pos
41-42 PreGMFlt3Neg
Erythrocyte/Megakaryocyte
43-44 CD9LoBEMP
45-46 CD9HiBEMP
47-48 PreCFUE
49-50 CFUE
51-52 MEP
53-55 EryBIPB
56-58 EryBIPO
GMP
59-60 GMP
61-62 GMP_IRF8hi
63-64 GMP_IRF8int
65-66 GMP_IRF8lo
67-71 GMPSiglecF
72-73 FcgRCD150
Basophil/Mast Cell
74-75 Mast
76-77 BasoBM
78 BasoSpl
Eosinophil
79-81 Eo
82-83 EoP
84-89 EoSSCHi
90-94 EoSSCLo
Neutrophil
95-96 NeutBM
97-100 NeutPB
Monocyte/Macrophage
101-102 Mac
103-104 InfMono
105-106 MonoBM
107-108 MonoPB
cDC
109-110 cDC2
In vivo pDC
111-112 pDC
B Cell
113-114 Bcell
NK Cell
115-116 NK
T Cell
117-118 CD4T
119-120 CD8T
121-122 EffCD4T
123-124 MemCD4T
125-126 MemCD8T
127-128 NveCD4T
129-130 NveCD8T
131-132 RegT
Hoxb8 MYB Leukemias
Monocytes Macrophages
B Cells
cDCs
In vivo pDCs
Hoxb8 pDCs
Hoxb8 MYB Leukemia
Cdkn2a-WT MYB::PLEKHO1
Cdkn2a-KO MYB::PLEKHO1
Cdkn2a-KO MYB-TR
Hoxb8 In Vitro
Hoxb8 pDC
In Vivo
B Cell
Monocyte/Macrophage
cDC
In vivo pDC
B
Genes bound by MYB::PLEKHO1 in
Cdkn2a-KO MYB::PLEKHO1 leukemia (n=500)
Cell Cycle
Metabolism of RNA
Gene expression (Transcription)
Metabolism of proteins
M Phase
Metabolism
Immune System
Post−translational protein modification
Generic Transcription Pathway
Cell Cycle Checkpoints
−log10(p)
Overlap
30
40
50
60
Overlap Ratio
0.07
0.06
0.05
0.04
C
V5 CUT&RUN: Cdkn2a-KO MYB::PLEKHO1 Leukemia
Motif Rank
-log10(p)
MYB 18.5%
IRF 43.1%
KLF 28.4%
n=1770
D
V5 signal at MYB motifs
-2kb
0
+2kb
Distance from motif

Figure S5: BPDCN MYB fusions bind to G2/M cell cycle genes in myeloid-dendritic leukemia.

(A) Principal component analysis of RNA-seq data from Hoxb8-FL cell-derived leukemias of the indicated genotypes, wild-type undifferentiated Hoxb8-FL cells and Hoxb8-FL cell-derived pDCs, and hematopoietic cell populations from the Haemopedia dataset (45). Lower panel shows the highlighted section zoomed in with points recolored to differentiate leukemia genotypes. (B) Top 10 ranked Reactome gene sets overlapping with the 500 unique genes bound by MYB::PLEKHO1 nearest to the transcription start site in *Cdkn2a*-KO MYB::PLEKHO1 leukemia cells. (C) Top 3 ranked motifs enriched in V5 CUT&RUN peaks in *Cdkn2a*-KO MYB::PLEKHO1 leukemia cells, and percent of peaks containing each motif. (D) Heatmap showing V5 CUT&RUN signal centered on MYB motifs inside V5 binding peaks located within 10 kb of a transcription start site, ordered by signal strength at the motif (n=2100 motifs).

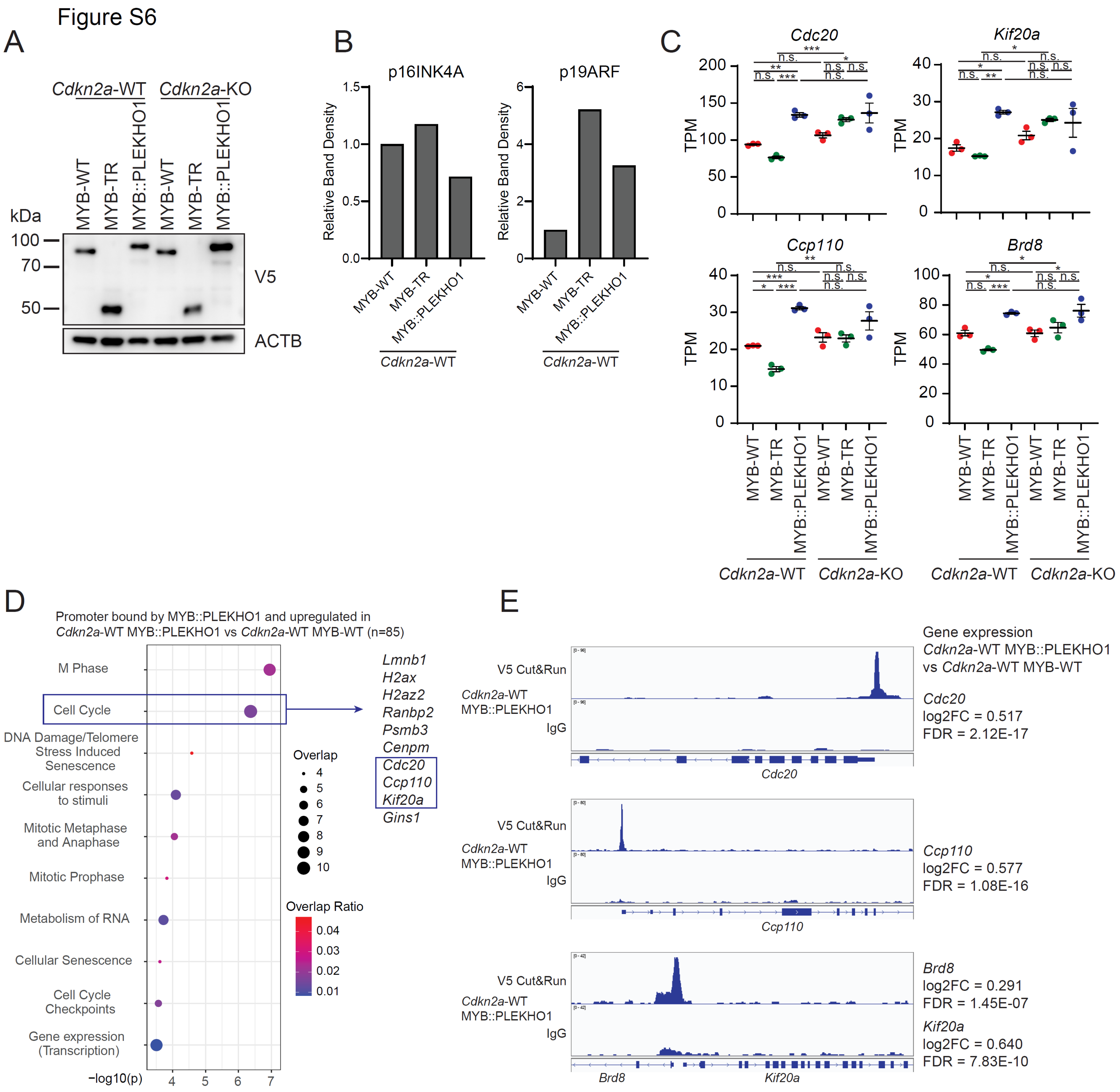

Figure S6: BPDCN MYB fusions bind to and activate G2/M cell cycle genes in Hoxb8-FL.MS5 cells.

(A) Western blot showing expression of V5-MYB constructs in Hoxb8-FL.MS5 cells of the indicated genotypes. (B) Quantification of western blot bands for p16INK4A and p19ARF stains shown in Figure 5D, normalized to ACTB signal and shown relative to MYB-WT. (C) TPM of *Cdc20*, *Ccp110*, *Kif20a* and *Brd8* in Hoxb8-FL.MS5 cells of the indicated genotypes (n=3 per genotype). Data represent mean ± SEM. Significance determined by one-way ANOVA with Tukey correction for multiple comparisons. * p<0.05, ** p<0.01, *** p<0.001. (D) Top 10 ranked Reactome gene sets overlapping with genes showing both increased expression in *Cdkn2a*-WT MYB::PLEKHO1 relative to *Cdkn2a*-WT MYB-WT Hoxb8-FL.MS5 and MYB::PLEKHO1 binding at promoter regions in *Cdkn2a*-WT MYB::PLEKHO1 Hoxb8-FL.MS5 cells (n=85). All overlapping genes in the "Cell Cycle" gene set (n=10) are shown. (E) V5 CUT&RUN tracks at the *Cdc20*, *Ccp110* and *Brd8*/*Kif20a* loci in *Cdkn2a*-WT MYB::PLEKHO1 Hoxb8-FL.MS5 cells. Gene expression fold changes and FDR values determined by DESeq2 are shown for *Cdkn2a*-WT MYB::PLEKHO1 Hoxb8-FL.MS5 cells relative to *Cdkn2a*-WT MYB-WT Hoxb8-FL.MS5 cells.

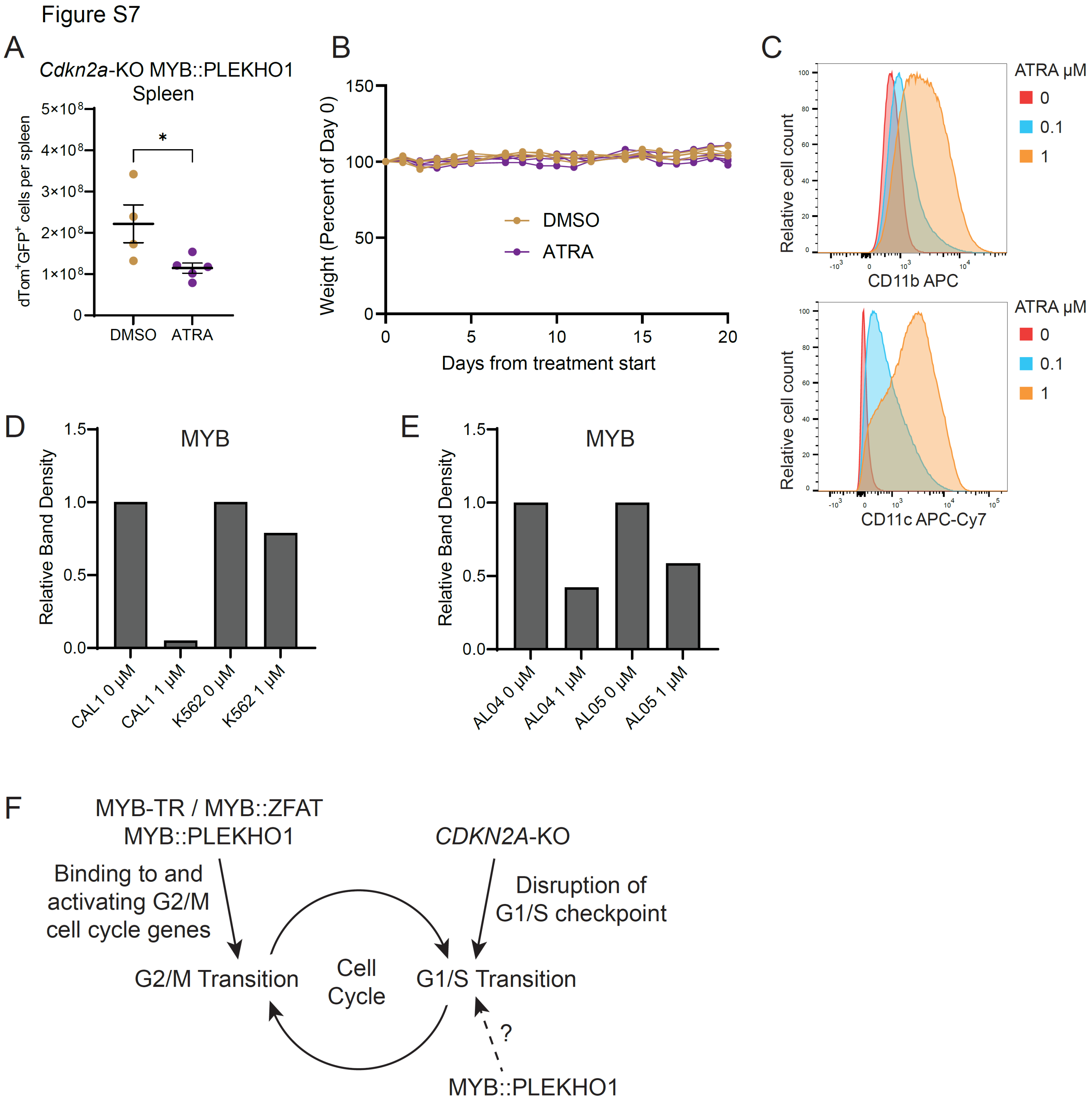

Figure S7: BPDCN cells undergo loss of MYB and cell death upon ATRA treatment.

(A) Number of viable GFP$^+$dTom$^+$ cells per spleen of *Cdkn2a*-KO MYB::PLEKHO1 secondary recipient mice treated daily with 20 mg/kg ATRA for 21 days (DMSO n=4, ATRA n=5). Data represent mean ± SEM. Significance determined by t test. * $p<0.05$. (B) Body weight during treatment period of mice in (A), shown as percentage of starting weight. (C) Representative fluorescence intensity histograms showing cell frequency relative to mode for CD11b APC and CD11c APC-Cy7 in CAL1 cells after 2 days of ATRA treatment at the indicated dose. (D) Quantification of western blot bands for MYB stain shown in Figure 6H, normalized to ACTB signal and shown relative to 0 µM ATRA. (E) Quantification of western blot bands for MYB stain shown in Figure 6I, normalized to ACTB signal and shown relative to 0 µM ATRA. (F) Model showing proposed mechanism of MYB fusion oncogenesis in BPDCN.
