# Supplementary material for: BPDCN MYB fusions regulate cell cycle genes, impair differentiation, and induce myeloid–dendritic cell leukemia
Source: JCI Insight. 2024 Dec 20;9(24):e183889. doi: 10.1172/jci.insight.183889 (PMC11665559; doi:10.1172/jci.insight.183889)

Figure 2B
ACTB stain

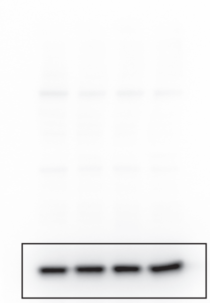

Figure 2B
GAPDH stain

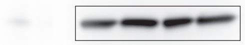

Figure 2B
MYB stain

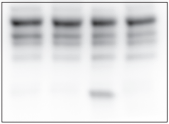

Figure 2B
V5 stain

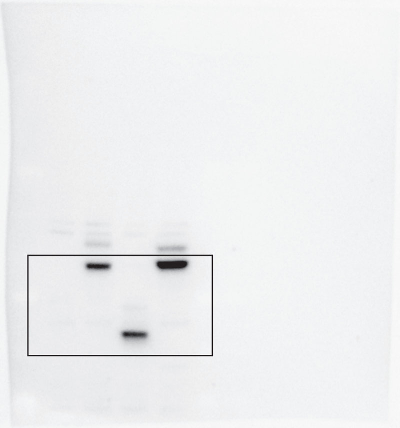

Figure 5D
ACTB stain (bottom)

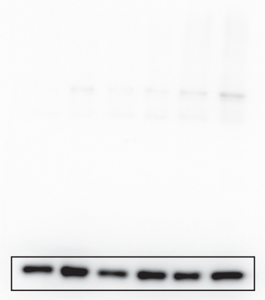

Figure 5D
ACTB stain (top)

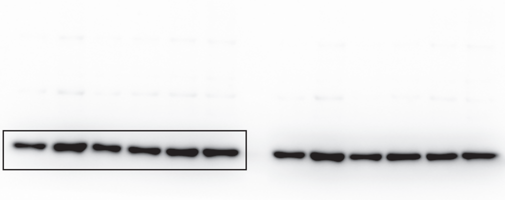

Figure 5D
p16INK4A stain

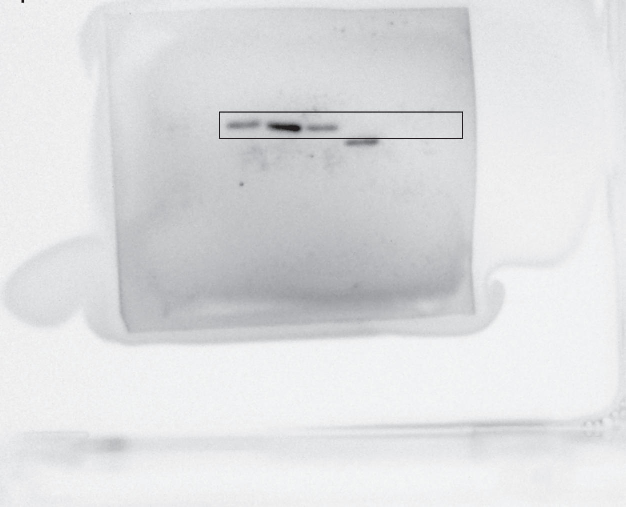

Figure 5D
p19ARF stain

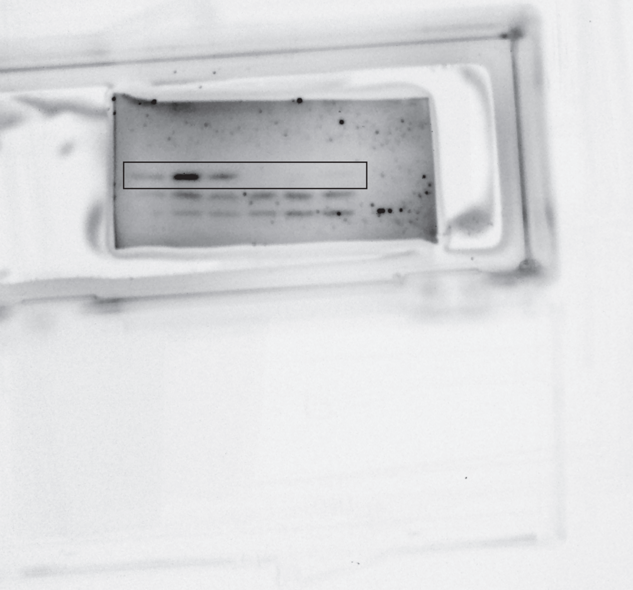

Figure 6H
ACTB stain

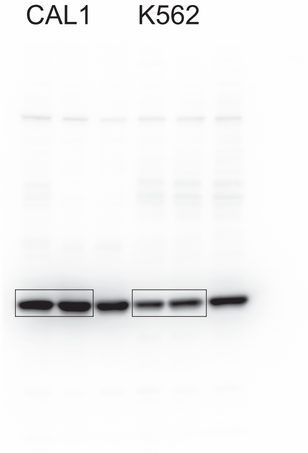

Figure 6H
MYB stain

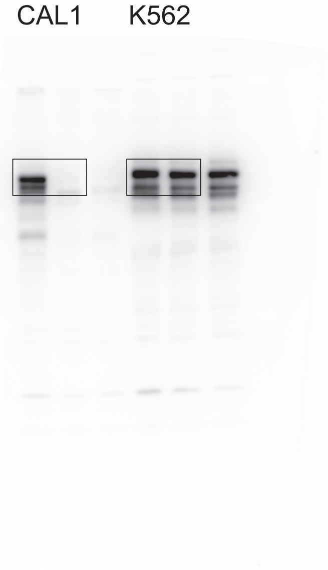

Figure 6I
ACTB stain

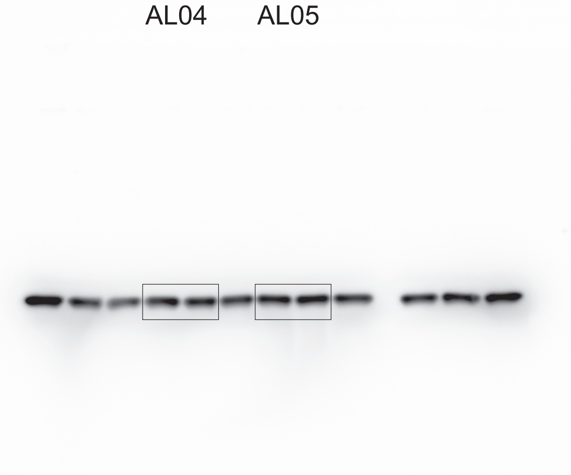

Figure 6I
MYB stain

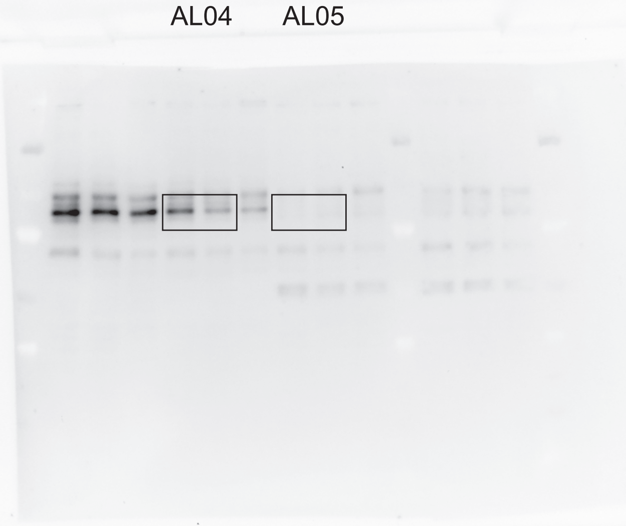

Figure 6I
MYB stain (MYB-ZFAT)

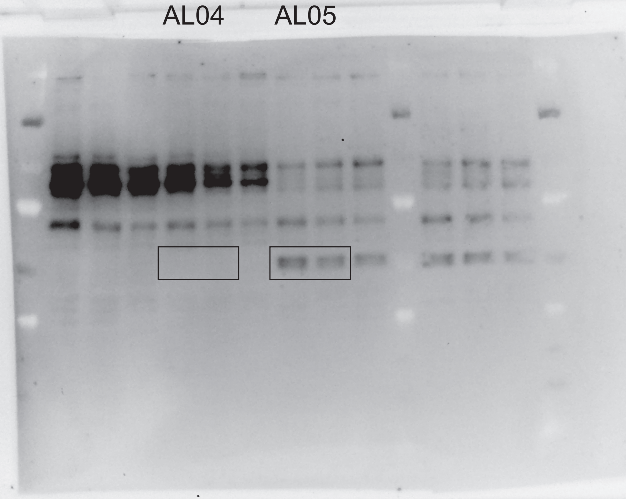

Figure S3A
GAPDH stain

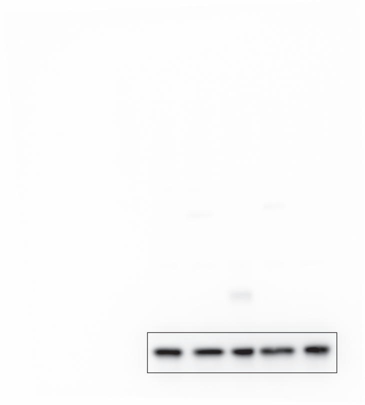

Figure S3A
V5 stain

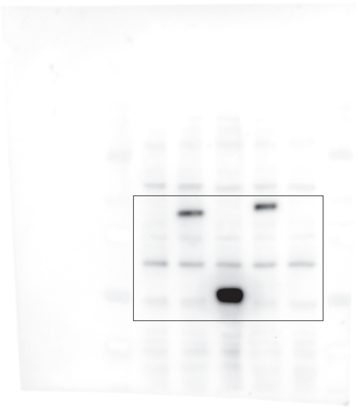

Figure S3C
ACTB stain

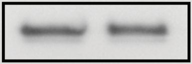

Figure S3C
p19ARF stain

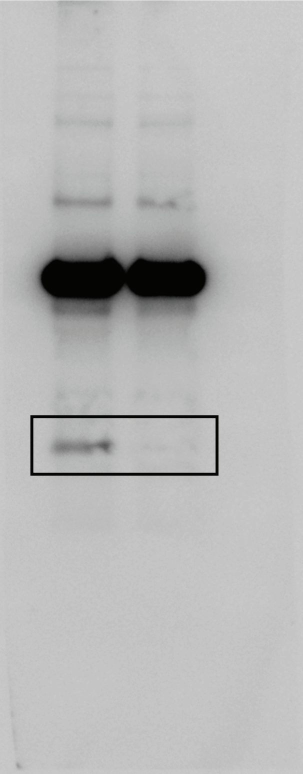

ACTB band

p19ARF band

Figure S4C
ACTB stain (left)

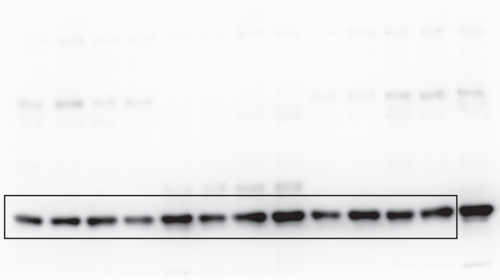

Figure S4C
ACTB stain (right)

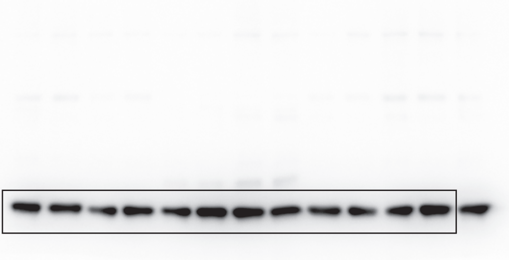

Figure S4C
MYB stain

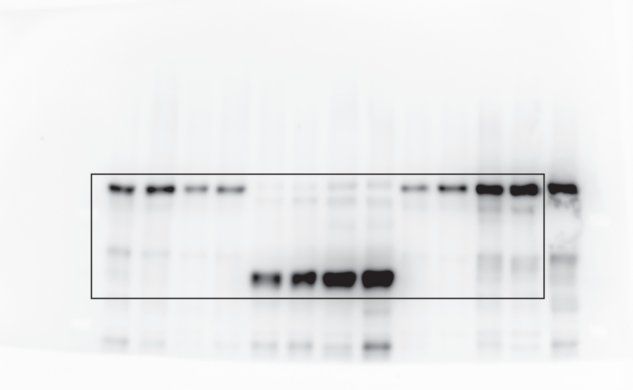

Figure S4C
p16INK4A stain

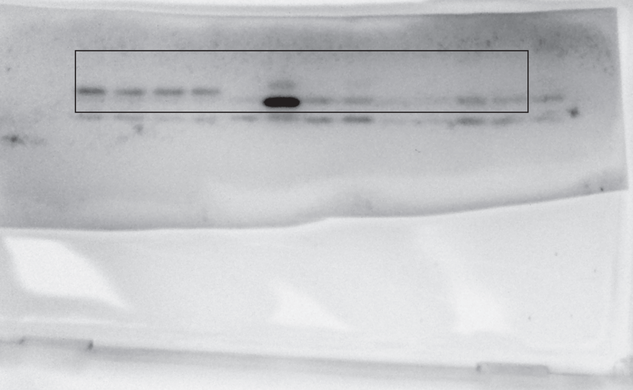

Figure S4C
p19ARF stain

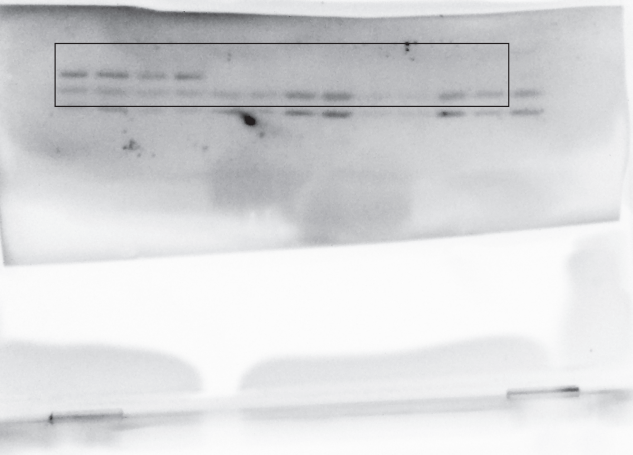

Figure S4C
V5 stain

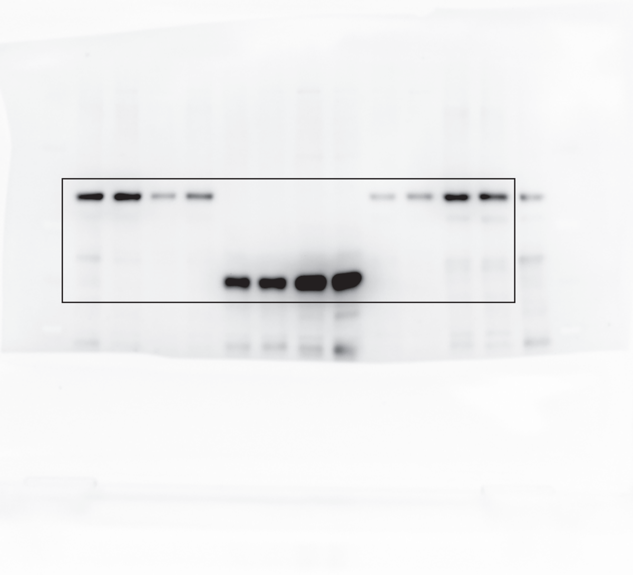

Figure S6A
ACTB stain

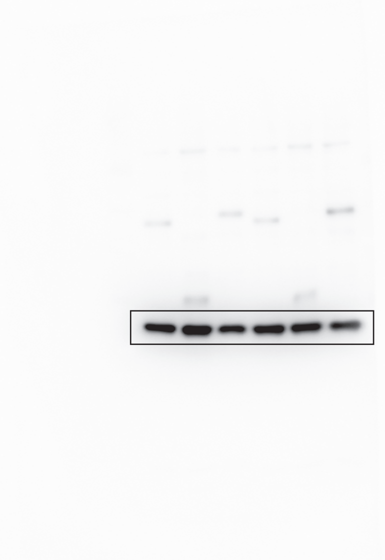

Figure S6A
V5 stain

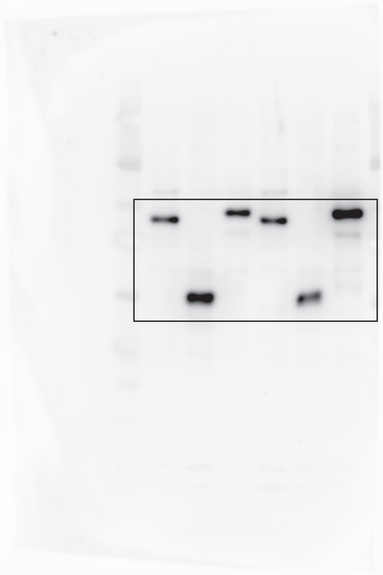

Supplement: Unedited blot and gel images [file jciinsight-9-183889-s129.pdf]
